# Quantitative Delineation of the Low Energy Decomposition Pathway for Lithium Peroxide in Lithium–Oxygen Battery

**DOI:** 10.1002/advs.202001660

**Published:** 2020-08-11

**Authors:** Arghya Dutta, Kimihiko Ito, Akihiro Nomura, Yoshimi Kubo

**Affiliations:** ^1^ Center for Green Research on Energy and Environmental Materials National Institute for Materials Science 1‐1 Namiki Tsukuba 305‐0044 Japan; ^2^ NIMS‐SoftBank Advanced Technologies Development Center National Institute for Materials Science 1‐1 Namiki Tsukuba 305‐0044 Japan

**Keywords:** energy efficiencies, energy storage, Li_2_O_2_ growth models, Li–O_2_ batteries, recharge mechanisms

## Abstract

Identification of a low‐potential decomposition pathway for lithium peroxide (Li_2_O_2_) in nonaqueous lithium–oxygen (Li–O_2_) battery is urgently needed to ameliorate its poor energy efficiency. In this study, experimental data and theoretical calculations demonstrate that the recharge overpotential (*η*_RC_) of Li–O_2_ battery is fundamentally dependent on the Li_2_O_2_ crystallization pathway which is intrinsically related to the microscopic structural properties of the growing crystals during discharge. The Li_2_O_2_ grown by concurrent surface reduction and chemical disproportionation seems to form two discrete phases that have been deconvoluted and the amount of Li_2_O_2_ deposited by these two routes is quantitatively estimated. Systematic analyses have demonstrated that, regardless of the bulk morphology, solution‐grown Li_2_O_2_ shows higher *η*_RC_ (>1 V) which can be attributed to higher structural order in the crystal compared to the surface‐grown Li_2_O_2_. Presumably due to a cohesive interaction between the electrode surface and growing crystals, the surface‐grown Li_2_O_2_ seems to possess microscopic structural disorder that facilitates a delithiation induced partial solution‐phase oxidation at lower *η*_RC_ (<0.5 V). This difference in *η*_RC_ for differently grown Li_2_O_2_ provides crucial insights into necessary control over Li_2_O_2_ crystallization pathways to improve the energy efficiency of a Li–O_2_ battery.

## Graphical abstract

Herein, a fundamental correlation between the lithium peroxide crystallization pathway and subsequent recharge mechanism is established by combination of theory and experiment. The lithium peroxide grown by concurrent disproportionation and surface reduction is found to form two coexisting phases. Quantitative deconvolution shows that the lithium peroxide grown on electrode surface decomposes at low overpotential by a partial solution phase oxidation.

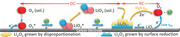

## 1 Introduction

Nonaqueous lithium–oxygen (Li–O_2_) battery, with a theoretical specific energy of ≈3.5 kWh kg^−1^, is considered to be one of the most promising energy storage technologies to cope with the ever‐increasing energy demand of the modern society for both mobile and stationary applications. The overall reversible electrochemical reaction in a nonaqueous Li–O_2_ battery involves a two‐electron (2e^−^) redox of oxygen (O_2_) in the presence of lithium ions (Li^+^) (2Li^+^ + O_2_ + 2e^−^ ↔ Li_2_O_2_(s); *E* ^0^ = 2.96 V vs Li/Li^+^) producing solid lithium peroxide (Li_2_O_2_) as the discharge (DC, oxygen reduction reaction (ORR)) product, which subsequently decomposes in the reverse reaction during recharge (RC, oxygen evolution reaction (OER)).^[^ ^1^ ^]^ Despite the promise of Li–O_2_ battery as a next generation storage system, low round‐trip energy efficiency (ratio of energy densities during DC and RC respectively) due to high RC overpotential (*η*, difference between thermodynamic (*E* ^0^) and measured (*E*) potential (*η* = |*E* − *E* ^0^|); usually *η*_RC_ > 1 V) and as a whole poorly understood RC mechanism pose a significant challenge before its realization.^[^ ^2^ ^]^ Although several electrocatalysts and soluble redox‐mediators were used to reduce the *η*_RC_, they could not truly improve the reversibility of oxygen‐electrochemistry, rather the solid catalysts exacerbated parasitic reactions and shuttling of redox‐mediators on the unprotected negative electrode led to passivation of Li metal.^[^ ^3^ ^]^ Moreover, use of an electrocatalyst seems redundant due to low kinetic overpotential for OER in Li–O_2_ battery as reported by density functional theory (DFT) calculations.^[^ ^4^ ^]^ Consistent with these theoretical studies, several experimental reports also found that the onset potential for Li_2_O_2_ oxidation is indeed very close to the equilibrium potential.^[^ ^5^ ^]^ However, despite the low kinetic overpotential for OER, the *η*_RC_ gradually increases during the course of RC and a two‐stage RC profile consisting of a low‐potential (<4 V vs Li/Li^+^) sloping region and a high‐potential (>4 V vs Li/Li^+^) flat plateau is usually observed for catalyst/redox‐mediator free Li–O_2_ batteries.^[^ ^6^ ^]^ The rise in *η*_RC_ has been qualitatively attributed to several interrelated factors such as physicochemical properties of the discharged Li_2_O_2_, stability of the electrode/electrolyte, presence of additives/impurities in the electrolyte etc.^[^ ^6^, ^7^ ^]^ Unfortunately, the effects from these factors have not been decoupled yet and combining results from independent experiments is often difficult due to completely different experimental conditions. As a result, contradictory reports explaining decomposition pathway of Li_2_O_2_, number of steps involved, reaction intermediates, kinetics of OER and exact reaction interface remain specific to a particular system and provide inconsistent understanding about the overall RC mechanism of Li–O_2_ battery.^[^ ^4^, ^6^, ^7^, ^8^ ^]^ Therefore, in order for realizing practical applications of Li–O_2_ battery by suppressing the increase of *η*_RC_, it is essential to identify a general low energy decomposition pathway for Li_2_O_2_.

Herein, we combine several in situ and ex situ experimental measurements with theoretical modelling to systematically decouple the crucial factors affecting the RC of Li–O_2_ battery and delineate a non‐catalytic low energy decomposition route for Li_2_O_2_. By detailed analysis we clearly demonstrate that RC potential of a Li–O_2_ battery fundamentally depends on the Li_2_O_2_ crystallization pathway which is intrinsically related to the microscopic structural properties of the growing Li_2_O_2_ crystals during DC of the cell. It is known that nucleation and growth of Li_2_O_2_ occurs via either chemical disproportionation or electrochemical reduction of intermediate reaction species LiO_2_.^[^ ^9^ ^]^ However, here we propose that there is no sharp partition between these two reaction pathways. Rather, depending on the thermodynamic feasibility, Li_2_O_2_ grows by concurrent electrochemical surface reduction and solution mediated chemical disproportionation reaction over a wide range of DC conditions. The Li_2_O_2_ grown by these two routes seems to form two coexisting discrete phases with subtle structural differences that result in distinctly different *η*_RC_. With the help of theory and experimental results we have quantitatively deconvoluted and estimated the amount of surface and solution grown Li_2_O_2_ during DC of the cells at different conditions. Unlike previous reports, here we clearly demonstrate that, no matter what the DC conditions, electrode/electrolyte properties and bulk morphology of Li_2_O_2_ are, solution‐grown Li_2_O_2_ always shows higher *η*_RC_ (>1 V) compared to that (<0.5 V) of surface‐grown Li_2_O_2_. The difference in *η*_RC_ can be attributed to intrinsic structural differences in Li_2_O_2_ crystals grown by two different routes. The higher *η*_RC_ of solution grown Li_2_O_2_ can be attributed to relatively higher structural order in Li_2_O_2_ crystals grown by chemical disproportionation. In contrast, due to a possible cohesive interaction between the electrode surface and growing crystals, the surface‐grown Li_2_O_2_ seems to possess microscopic structural disorder and improved charge transport properties that facilitate a delithiation induced partial solution‐phase oxidation at relatively lower *η*_RC_ (<0.5 V). Our observations suggest that ensuring surface‐growth of Li_2_O_2_ by strategic modification of electrode and proper choice of electrolyte can simultaneously provide a large DC capacity with high RC efficiency to mitigate the capacity‐rechargeability trade‐off in Li–O_2_ battery.

## 2 Results and Discussions

### 2.1 Discharge Rate Dependent Recharge Behavior of Li–O_2_ Batteries

**Figure 1:**
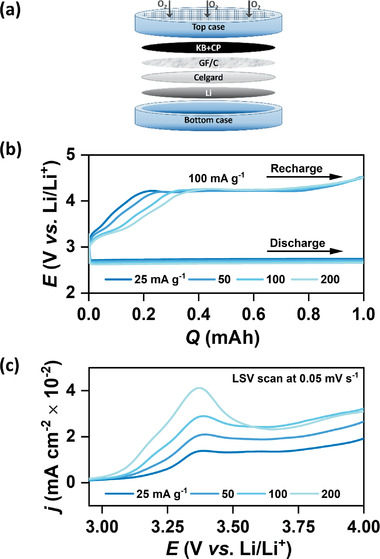
a) Schematic representation of a Li–O_2_ coin cell with Ketjenblack (KB) as the positive electrode. b) Potential (*E*) versus capacity (*Q*) plot for KB electrode in LiTFSI/TEGDME electrolyte (<20 ppm H_2_O). The cells are discharged at different current densities (*j*_DC_) but recharged at the same current density (*j*_RC_) of 100 mA g^−1^. c) Anodic linear sweep voltammetric (LSV, 0.05 mV s^−1^) curves after discharge at different *j*_DC_ up to the fixed capacity (*Q*_DC_) of 1 mAh.

Coin type CR2032 cells were assembled using Ketjenblack (KB) as the positive electrode prepared by KB‐slurry (in an *N*‐methyl‐2‐pyrrolidone (NMP) solvent with 20 wt% LITHion (Ion Power) as the binder) pasted on Toray carbon paper (CP, TGP‐H‐30, 1.6 cm diameter), 1 m lithium bis(trifluoromethanesulfonyl)imide (LiTFSI) in tetraethylene glycol dimethyl ether (TEGDME or tetraglyme; <20 ppm H_2_O by Karl Fischer titration, unless otherwise mentioned) and lithium foil (Li, 1.6 cm diameter) as the negative electrode. TEGDME has been chosen as the representative solvent due to its acceptable stability in Li–O_2_ battery, good compatibility with Li metal and intermediate value of the Gutmann donor number (16.6 kcal mol^−1^) that is known to be a major solvent property influencing the ORR and OER mechanisms.^[^ ^10^ ^]^ The configuration of a typical Li–O_2_ coin cell is schematically shown in **Figure** **1**a and the detailed cell‐assembly procedure is provided in the Supporting information Experimental section.

Figure 1b shows the first cycle of galvanostatic DC/RC of Li–O_2_ cells with KB electrodes where DC of the cells is carried out at four different current densities (*j*_DC_) 25, 50, 100, and 200 mA g^−1^ up to a fixed capacity (*Q*) of 1 mAh. On the contrary, all the cells are recharged at 100 mA g^−1^ with cut‐off conditions of 100% RC or 4.5 V versus Li/Li^+^. DC/RC currents for all the cells are normalized to the mass of KB (≈0.5±0.05 mg cm^−2^) in the electrode. During discharge, all the cells show a nearly constant‐potential plateau in the DC curve with gradual increase in average *η*_DC_ from 223 mV at 25 mA g^−1^ to 316 mV at 200 mA g^−1^. A magnified view of the DC curves in the range of 0.4–0.6 mAh is shown in Figure S1 in the Supporting Information. This increase in average *η*_DC_ with *j*_DC_ is characteristic of *i*R drop due to buildup of insulating solid Li_2_O_2_ (having ionic and electronic conductivities in the range of 10^−19^–10^−20^ S cm^−1^ at 25 °C) during DC of the cells.^[^ ^4^, ^11^ ^]^ In contrast, despite recharged at the same current density (*j*_RC_), all the cells show more pronounced difference in RC behavior. Qualitatively, all the RC curves consist of two stages; an initial sloping region with gradual increase in *η*_RC_ usually up to 20–40% state of charge (SOC) followed by a flat high‐voltage plateau above 4 V versus Li/Li^+^. From Figure 1b, it is apparent that the fraction of charge passed in the sloping region or in other words, the amount of Li_2_O_2_ decomposed at low *η*_RC_ (<1 V) depends on the DC current. Nonetheless, the potential in the plateau region remains quite similar. In addition to galvanostatic RC, we have also recharged the cells by anodic linear sweep voltammetry (LSV, sweep rate 0.05 mV s^−1^) from 2.95 to 4.7 V versus Li/Li^+^ after galvanostatic DC at different *j*_DC_ up to a *Q*_DC_ of 1 mAh. The LSV curves over the full range of potential are provided in Figure S2 in the Supporting Information, whereas a magnified section from 2.95 to 4 V versus Li/Li^+^ is shown in Figure 1c. Two distinctly different oxidation peaks, one centered at ≈3.35 V versus Li/Li^+^ and the other at >4.2 V versus Li/Li^+^, are observed for all the cells. Consistent with the trend in sloping region of galvanostatic curves, the intensity of the LSV peak at ≈3.35 V versus Li/Li^+^ also increases with increase in *j*_DC_. These observations clearly show that larger fraction of Li_2_O_2_ deposited at relatively higher *j*_DC_ can be decomposed at lower *η*_RC_.

### 2.2 Morphological and Structural Analysis of Rate Dependent Discharge Products

**Figure 2:**
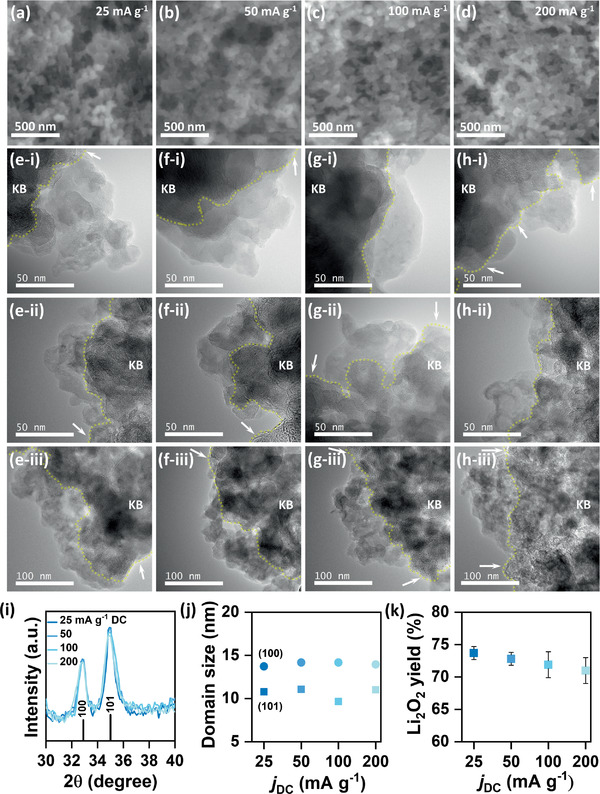
a–d) Scanning electron microscopy (SEM) images of KB electrodes after discharge at different *j*_DC_ up to a *Q*_DC_ of 1 mAh in LiTFSI/TEGDME electrolyte (<20 ppm H_2_O). e–h) Transmission electron microscopy (TEM) images and i) X‐ray diffraction (XRD) patterns of the same electrodes. All the XRD data are normalized to the intensity of KB+CP peak at 2*θ* = 54.3. j) Plot of domain size versus *j*_DC_ for both (100) and (101) reflections of Li_2_O_2_. Domain size is calculated by using Scherrer equation. k) Plot of Li_2_O_2_ yield measured by titration with TiOSO_4_ solution after DC at different *j*_DC_. TEM images marked as (i–iii) represent three different locations of the same electrode at respective *j*_DC_. The white arrows indicate parts of carbon surface not covered with Li_2_O_2_. The Yellow dotted lines in (e–h) demarcate the Li_2_O_2_/carbon interface. XRD peak positions for standard Li_2_O_2_ sample are shown by black vertical lines at the bottom of (i). The vertical lines in (k) represent the error range of measured data from three sets of experiment.

A nanoscale continuum model for the growth of Li_2_O_2_ crystals in Li–O_2_ battery predicted a transition from large toroids (at lower discharge rates) to thin‐film/nanoparticles (at higher discharge rates) at a critical *j*_DC_ implying that large toroids and thin‐film/nanoparticles cannot coexist in the DC product.^[^ ^12^ ^]^ Consistent with this hypothesis, the DC current dependent RC profile of Li–O_2_ battery was previously attributed to morphological differences observed in Li_2_O_2_ deposited at different *j*_DC_.^[^ ^6b,c^ ^]^ These studies demonstrated that thin‐film/nanoparticle‐like Li_2_O_2_ formed at higher *j*_DC_ can be decomposed at lower *η*_RC_ sloping region, whereas large toroidal Li_2_O_2_ formed at lower *j*_DC_ gets decomposed at higher *η*_RC_ (>1 V) flat plateau.^[^ ^6b,c^ ^]^ However, it is not understood why a two‐stage RC profile, which according to those reports should be indicative of a mixed morphology in deposited Li_2_O_2_, is observed over a wide range of *j*_DC_. Moreover, a later study has evinced that a sufficiently dry glyme‐ether based electrolyte is not expected to show any *j*_DC_ dependent morphological differences in the discharged Li_2_O_2_, rather a film‐like Li_2_O_2_ is expected at all *j*_DC_.^[^ ^7c^ ^]^ As a result, sharp correlation between *j*_DC_‐dependent Li_2_O_2_ morphology and subsequent RC profile seems ambiguous. These contradictions in reported data lead to inconsistency in understanding the DC/RC process and ask for careful reconsideration of all the qualitative and quantitative parameters that affect the operation of Li–O_2_ battery. Besides, combining results from different independent experiments to provide a complete idea about DC/RC processes is often difficult due to high sensitivity of Li–O_2_ electrochemistry towards variation in experimental conditions.^[^ ^13^ ^]^ Therefore, here we have carried out several in situ and ex situ analyses under the same experimental set up inside a super‐dry room (maximum dew point −60 °C) in order to maintain similar effects by external factors in all of our experiments. The scanning electron microscopy (SEM) images in **Figure** **2**a–d show the morphology of the electrodes after DC at four different *j*_DC_. Possibly due to low water content (<20 ppm H_2_O by Karl Fischer titration) in our tetraglyme based electrolyte, no large particle/toroidal Li_2_O_2_ is observed at any current density from 25 to 200 mA g^−1^. In fact, all the electrodes look indistinguishable in the SEM images after DC up to a fixed *Q*_DC_ of 1 mAh. A closer look at the DC products by transmission electron microscopy (TEM), shown in Figure 2e–h, indicates that at all *j*_DC_ the deposited Li_2_O_2_ is composed of small nano‐sized conglomerated particles (30–40 nm diameter) that appear to be a film at low magnification. Another important revelation from TEM images is that the deposition is not conformal, rather particles of Li_2_O_2_ are nonuniformly deposited as islands leaving uncovered carbon surfaces around the deposits (bare carbon is pointed by white arrows in Figure 2e–h). Considering very small measurement area of TEM imaging, we have provided TEM images at three different locations of the same electrodes, marked as (i) to (iii) in Figure 2e–h, to show extensive nonuniformity of Li_2_O_2_ deposition throughout the electrode. The SEM and TEM images of pristine KB samples are also shown in Figure S3a,b in the Supporting Information, respectively, for comparison with the discharged electrodes. However, despite nonuniform deposition, holistically the average thickness of the deposited Li_2_O_2_ film decreases with increase in *j*_DC_. From these morphological analyses it is clear that the two‐stage RC profile and its dependence on *j*_DC_ in Li–O_2_ battery cannot be simply explained by the bulk morphologies of Li_2_O_2_. Crystallinity of Li_2_O_2_ is another important factor that has been considered by several groups to explain *j*_DC_ dependent RC profile of Li–O_2_ battery.^[^ ^6b,c^ ^]^ Operando X‐ray diffraction (XRD) studies during RC observed a non‐linear decay of peak intensity for Li_2_O_2_ where in the beginning, the peak intensity decays very slowly followed by a rapid decay at high RC potential.^[^ ^14^ ^]^ The slow decay in XRD peak intensity gives an indirect evidence for decomposition of Li_2_O_2_ with smaller domain size (or nano‐sized/amorphous Li_2_O_2_) at low RC potential, whereas the sharp decay directly indicates disappearance of larger domains of Li_2_O_2_ (or toroidal/crystalline Li_2_O_2_) at flat high‐voltage plateau. This explanation of the two‐stage RC profile seems quite reasonable since higher electronic as well as ionic conductivities of amorphous Li_2_O_2_ make it feasible to decompose at low *η*_RC_, whereas crystalline Li_2_O_2_ that behaves like an insulator decomposes at high voltage plateau.^[^ ^5^, ^15^ ^]^ However, exact mechanism of simultaneous deposition of amorphous and crystalline Li_2_O_2_ at a particular DC condition is not understood. Moreover, *j*_DC_ dependent variation in Li_2_O_2_ domain size is also questionable in a sufficiently dry ether‐based electrolyte.^[^ ^7c^ ^]^ Figure 2i and Figure S4 (Supporting Information) show the XRD patterns of the electrodes discharged at different current densities from 25 to 200 mA g^−1^. Superposition of peaks for both (100) and (101) reflections of Li_2_O_2_ discharged at all current densities is quite evident from the XRD patterns. The average domain sizes of Li_2_O_2_ discharged at four different *j*_DC_ vary in the ranges 13.7–14.2 and 9.7–11.1 nm for (100) and (101) reflections respectively as displayed in Figure 2j. It is necessary to mention that no correlation between *j*_DC_ and domain size can be established from these results due to experimental uncertainty over the small differences in full width half maximum (FWHM) values for corresponding peaks of the four samples. However, an apparent difference in domain size (≈3 nm) between (100) and (101) reflections for all the samples indicates anisotropy in the crystal structure of deposited Li_2_O_2_.^[^ ^14^ ^]^ An important corollary of the XRD data shown in Figure 2i,j is that it is difficult to discriminate the crystallinity of Li_2_O_2_ deposited at different *j*_DC_. Having said that, we do not disregard the effects of Li_2_O_2_ crystallinity on the RC profile of Li–O_2_ battery, we rather emphasize the limitation of ex situ XRD analysis in identifying subtle differences in *j*_DC_ dependent structural features of electrochemically formed Li_2_O_2_.^[^ ^14a^ ^]^ Instability of carbon electrode and nonaqueous liquid electrolyte towards reduced oxygen species is a serious problem in Li–O_2_ battery leading to buildup of insulating solid parasitic products that increase the impedance of the cell.^[^ ^16^ ^]^ As the cell‐impedance rises, decomposition of Li_2_O_2_ should require high *η*_RC_. In order to investigate any effect of parasitic side reactions on the *j*_DC_ dependent RC profile of Li–O_2_ battery, we have quantified the amount of Li_2_O_2_ after DC at different *j*_DC_ up to a fixed *Q*_DC_ of 1 mAh by titrating the electrodes with titanium (IV) oxysulfate (TiOSO_4_) solution. The yields of Li_2_O_2_ after DC have been shown in Figure 2k and are found to be 74 ± 1, 73 ± 1, 72 ± 2, and 71 ± 2% at *j*_DC_ 25, 50, 100, and 200 mA g^−1^ respectively. Lower than theoretical yield of Li_2_O_2_ is ascribed to parasitic reactions as mentioned above and the Fourier transform infrared spectrum (FTIR) of the KB electrode discharged at 100 mA g^−1^ in Figure S5 in the Supporting Information shows the existence of lithium carbonate (Li_2_CO_3_) and lithium carboxylates (RCO_2_Li) as the parasitic products. It has to be mentioned here that the parasitic products may have detrimental effect in increasing the *η*_RC_ of the cells but quite similar yield of Li_2_O_2_ (hence similar extent of parasitic reactions) after DC at different *j*_DC_ should exhibit comparable effects on *η*_RC_.

The structural, morphological and quantitative analyses demonstrated above could only point out a *j*_DC_ dependent difference in average thickness of the deposited Li_2_O_2_. A possible correlation between *η*_RC_ and thickness of Li_2_O_2_ deposit was reported earlier for a flat electrode and it was argued that relatively thinner parts of Li_2_O_2_ film could be decomposed at low *η*_RC_.^[^ ^5b^ ^]^ However, interestingly, the positions of anodic LSV peaks (in the ranges of <3.5 and >4 V vs Li/Li^+^) of a Li_2_O_2_ film of <3 nm thickness grown on a flat electrode resemble the positions of the LSV peaks for the oxidation of several tens of nanometer thick Li_2_O_2_ deposited on KB electrode as well as on other high surface area carbons reported previously.^[^ ^5^, ^17^ ^]^ Therefore, exact correlation between RC profile and Li_2_O_2_ thickness seems inconclusive. Besides, the stochasticity of Li_2_O_2_ layer thickness due to nonuniform deposition also makes it difficult to establish a quantitative relationship between thickness and RC potential. Nonetheless, based on the observation of nonuniform deposition, we have modelled the nucleation‐and‐growth of Li_2_O_2_ in our system in order to provide a quantitative insight about the relation between *j*_DC_ dependent Li_2_O_2_ deposition and subsequent RC profile.

### 2.3 Theoretical Modelling of Li_2_O_2_ Growth During Discharge

The DC of Li–O_2_ battery is known to involve multiple steps of ORR in nonaqueous conditions^[^ ^8c^ ^]^

(1) $$\begin{array}{c} {O_{2}}^{*}+Li^{+}+e^{-}\to \mathrm{Li}{O_{2}}^{*} \end{array}$$

(2) $$\begin{array}{c} \mathrm{Li}{O_{2}}^{*}↔\mathrm{Li}O_{2}\left(\mathrm{sol}.\right) \end{array}$$

(3) $$\begin{array}{c} \mathrm{Li}{O_{2}}^{*}+Li^{+}+e^{-}\to Li_{2}O_{2}\left(s\right) \end{array}$$

(4) $$\begin{array}{c} 2\mathrm{Li}O_{2}\left(\mathrm{sol}.\right)\to Li_{2}O_{2}\left(s\right)+O_{2} \end{array}$$

(Here the asterisk (*) represents the species adsorbed on the electrode surface)

**Figure 3:**
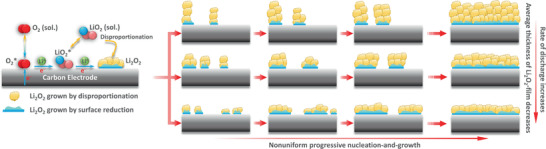
Schematic representation of a nonuniform nucleation‐and‐growth model presented in this work.

Based on these reaction steps we propose a nucleation‐and‐growth mechanism that agrees with the nonuniform deposition of Li_2_O_2_ as observed in the TEM images. In our model, reaction (1), i.e., formation of surface bound LiO_2_* is regarded as the first step of ORR. This ORR step is followed by concurrent nucleation (reaction (3)) and growth (reaction (4)) of Li_2_O_2_ on the KB surface. Negative free energies for both surface reduction and solution phase disproportionation indicate that simultaneous occurrence of these two reactions is thermodynamically feasible.^[^ ^6c^ ^]^ Here we specify that nucleation refers to surface‐growth that accounts for the coverage of Li_2_O_2_ layer on the electrode surface, whereas growth means precipitation of Li_2_O_2_ from solution phase on top of the nucleation sites. Li_2_O_2_ layer with thickness >10 nm (maximum electron tunneling distance through Li_2_O_2_ layer) observed in TEM images also indicates involvement of solution mediated vertical growth of Li_2_O_2_ in our cells.^[^ ^18^ ^]^ The solubility, if any, of intermediate LiO_2_ in sufficiently dry LiTFSI/TEGDME electrolyte (<20 ppm H_2_O) has been verified by cathodic LSV using rotating ring disk electrode (RRDE, rotation speed of 900 rpm) under 3‐electrode set‐up. From the LSV data shown in Figure S6 in the Supporting Information, a pronounced reduction current is observed at the KB disk below 2.76 V versus Li/Li^+^ and simultaneously an oxidizing current is observed at the GC ring held at 3.5 V versus Li/Li^+^. The reduction current corresponds to ORR at the KB disk while the ring current originates from the oxidation of solvated LiO_2_ (sol.) that diffuses from the disk to the ring due to the rotation of the electrode. The emergence of ring current confirms the solubility, at least partially, of LiO_2_ in dry LiTFSI/TEGDME electrolyte (<20 ppm H_2_O) and the results are consistent with previously reported studies.^[^ ^9^ ^]^ Higher binding energy of lithium as well as of oxygen leading to preferential nucleation at randomly distributed defect sites results in nonuniform nucleation of Li_2_O_2_ on carbon surface. Density functional theory (DFT) calculations showed that the adsorption energies at the defect sites can be higher by ≈1.5 and ≈4.4 eV for lithium and oxygen respectively compared to a perfect defectless site.^[^ ^19^ ^]^ KB indeed manifests considerable defects and consists of oxygen functional groups that can act as the adsorption sites for both oxygen and lithium. The Raman spectrum of KB in Figure S7 in the Supporting Information shows D‐band (*I*_D_, representative of disorder) to G‐band (*I*_G_, graphitic structure) intensity ratio (*I*_D_/*I*_G_) of 1.06 that certainly confirms presence of defects in the carbon structure.^[^ ^17^ ^]^ X‐ray photoelectron spectroscopic (XPS) analysis of the KB surface estimates oxygen to carbon ratio (O/C) of 0.027 where oxygen functionalities are identified to be C—O (e.g., phenol, ether, epoxy etc. at C1s ≈ 286.7 eV and O1s ≈ 533.5 eV), C=O (e.g., carbonyl, quinone etc. at C1s 287.5 eV and O1s 531.8 eV) and —COO^−^ (e.g., carboxylic acid, ester, lactone, etc. at C1s 289.3 eV) groups that are shown by deconvoluted XPS data in Figure S8 in the Supporting Information and the physicochemical properties of KB are summarized in Table S1 in the Supporting Information.^[^ ^20^ ^]^ According to our proposed mechanism, nucleation of Li_2_O_2_ starts preferentially at the defect sites of KB electrode and concurrent nucleation‐and‐growth occurs progressively until complete coverage of Li_2_O_2_ layer on the electrode surface. A schematic representation of the proposed DC mechanism is shown in **Figure** **3**.

In order to model the nucleation‐and‐growth mechanism described above, we have considered the kinetic equations describing the rates of reactions (1) (*r*_1_, mol m^−2^ s^−1^) and (3) (*r*_2_, mol m^−2^ s^−1^)

(5) $$\begin{array}{c} r_{1}=\;k_{1}c_{O_{2}}\exp\left(-\frac{\alpha_{1}F}{RT}\;\eta_{1}\right) \end{array}$$

(6) $$\begin{array}{c} r_{2}=\;k_{2}c_{LiO_{2}}\exp\left(-\frac{\alpha_{2}F}{RT}\;\eta_{2}\right) \end{array}$$

where *k*_1_ (m s^−1^) and *k*_2_ (s^−1^) are the rate constants for reduction of oxygen (O_2_) and surface adsorbed lithium superoxide (LiO_2_*) respectively, $c_{O_{2}}$ (mol m^−3^) and $c_{\mathrm{Li}O_{2}}$ (mol m^−2^) are the concentrations of oxygen in the electrolyte and surface concentration of LiO_2_* on the electrode respectively, *α*_1_ and *α*_2_ are the charge transfer symmetry factors approximated to be 0.5, *η*_1_ and *η*_2_ are the overpotentials for the respective steps assumed to be *η*_1_ ≈ *η*_2_ = *η*_DC_. *F* (96 485 C mol^−1^), *R* (8.314 J mol^−1^ K^−1^), and *T* (298 K) have their usual meaning. The solvation kinetics of LiO_2_* is represented by

(7) $$\begin{array}{c} r_{s}=\;k_{s}c_{\mathrm{Li}O_{2}} \end{array}$$

where *r*_s_ (mol m^−2^ s^−1^) is the solvation rate of LiO_2_* and *k*_s_ (s^−1^) is the solvation rate constant. These equations show that unlike nucleation rate (*r*_2_), which depends on the DC overpotential (*η*_DC_), the growth rate depends on the rate of LiO_2_* solvation (*r*_s_) and its supersaturation in the electrolyte. For simplicity, here we consider same rates of reactions at the superficial surface and also inside the bulk of the porous electrode. We further assume that since nucleation occurs by surface reduction, the nucleating layer on the electrode surface can have a maximum average thickness (*h*_0_) limited by electron tunneling length and based on this assumption the areal growth rate of Li_2_O_2_ can be described as

(8) $$\begin{array}{ccc} \frac{dA_{Li_{2}O_{2}}}{dt}=A_{\mathrm{electrode}}\;\; & \times & \;\frac{M_{Li_{2}O_{2}}}{\rho_{Li_{2}O_{2}}\;\times \;h_{0}}\;\times \;c_{O_{2}}\;\\ & & \times \;\frac{k_{1}\;\times \;\exp\left(-\frac{2\alpha F}{RT}\eta_{DC}\right)}{\exp\left(-\frac{\alpha F}{RT}\eta_{DC}\right)\;+\;\frac{k_{s}}{k_{2}}} \end{array}$$

where *A*_electrode_ is the electrochemically active surface area (ECSA, 0.028 m^2^) of the KB electrode (measured by cyclic voltammetry (CV, Figure S9, Supporting Information) of a symmetric KB|KB cell and the calculation is shown in the supporting information), $M_{Li_{2}O_{2}}$ and $\rho_{Li_{2}O_{2}}$ are the molar mass (45.88 g mol^−1^) and bulk density (2.31 × 10^6^ g m^−3^) of Li_2_O_2_ respectively and as previously mentioned, *h*_0_ is the maximum average thickness of the nucleating layer. Full derivation of Equation (8) is provided in the supporting information. According to this equation, sudden‐death during DC (or full saturation of electrode surface) occurs at a time (*t*_s_) when $A_{Li_{2}O_{2}}=\;A_{\mathrm{electrode}}$. Figure S10 in the Supporting Information shows DC of the cells at four *j*_DC_ from 25 to 200 mA g^−1^ up to the cut‐off potential of 2 V versus Li/Li^+^. A sharp decay in DC potential up to 2 V versus Li/Li^+^ (sudden‐death) is observed at the end of DC for all the cells in Figure S10 in the Supporting Information. This sudden‐death denotes the complete coverage of the electrode surface by insulating Li_2_O_2_ as the DC product. Had the sudden death been due to charge transport limitation through a conformal layer of Li_2_O_2_, as observed in flat electrodes, the *Q*_DC_ would be inversely proportional to *j*_DC_.^[^ ^21^ ^]^ Figure S11 in the Supporting Information shows a plot of *Q*_DC_ versus *j*_DC_ for the KB electrodes where the inverse relationship, shown by the gray solid line, significantly deviates from the experimental data points.

### 2.4 Quantitative Correlation between Li_2_O_2_ Growth Mechanism and Recharge Profile

**Figure 4:**
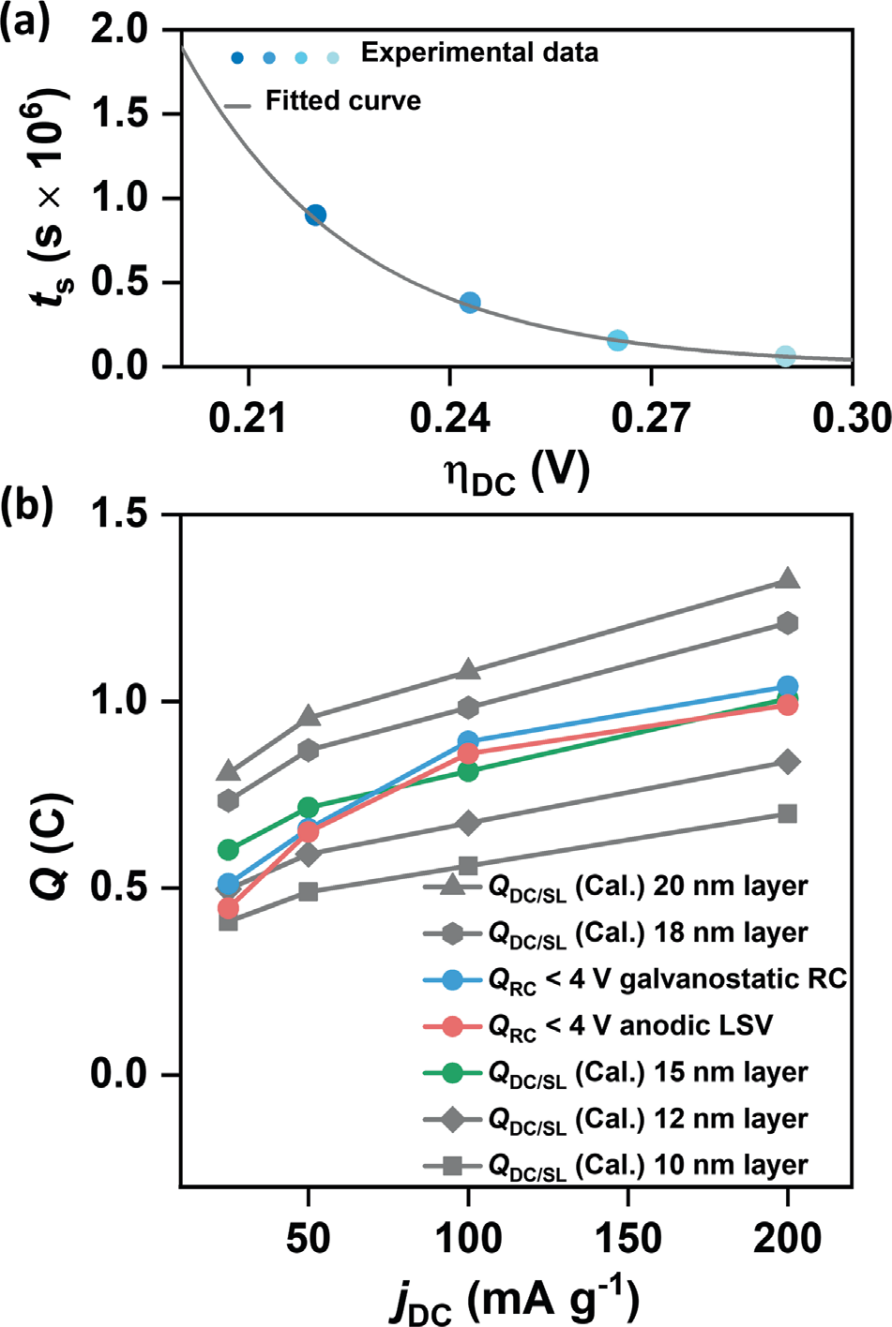
a) Plot of the time (*t*_s_) required for galvanostatic full discharge (up to 2 V vs Li/Li^+^) of a cell versus DC overpotential (*η*_DC_) using KB as the electrode and LiTFSI/TEGDME as the electrolyte (<20 ppm H_2_O) and fitting of this experimental data based on the nucleation and growth model presented in this work. b) Plot of the amount of charge (*Q*_RC_) passed during RC below 4 V versus Li/Li^+^ and the calculated charge (*Q*_DC/SL_) stored as Li_2_O_2_ layer with 10, 12, 15, 18, and 20 nm thickness grown by surface reduction against the respective current densities. For all the cases in (b) a *Q*_DC_ of 1 mAh is considered.

We have plotted the *t*_s_ versus *η*_DC_ for the cells discharged at *j*_DC_ from 25 to 200 mA g^−1^ in **Figure** **4**a. Values of *η*_DC_ for different *j*_DC_ are obtained by subtracting the equilibrium potential (*E* ^0^) of 2.96 V versus Li/Li^+^ from the DC potentials (*E*) in Figure 1b at 0.5% state of DC. Although the maximum tunneling length for a compact Li_2_O_2_ layer is reported to be ≈10 nm, presence of defects and grain boundaries can increase the tunneling length.^[^ ^11^, ^18^ ^]^ Therefore, to fit the experiemntal data in Figure 4a by using Equation (8) and for subsequent calculations we have considered different *h*_0_ values in the range 10–20 nm. A representative fitting of the experimental data points considering *h*_0_ value of 15 nm in Equation (8) is shown by the solid gray line in Figure 4a. The numerical values for all the fitting parameters used in Figure 4a are provided in **Table** **1**. Using the values of the fitting parameters in the *t*_s_ versus *η*_DC_ plots, next, we have computed the area covered by the Li_2_O_2_ layer discharged at different *j*_DC_ up to a limited *Q*_DC_ of 1 mAh. In order to correct the effect of overlapping Li_2_O_2_ particles, Kolmogorov's phase transformation theory is applied to calculate the fractional area coverage of the electrode by Li_2_O_2_ layer at a given time frame

(9) $$\begin{array}{c} \theta_{Li_{2}O_{2}}=\;1-\exp\left(-\frac{A_{Li_{2}O_{2}}}{A_{\mathrm{electrode}}}\right) \end{array}$$

**Table 1:** Physical constants and fitting parameters

$M_{Li_{2}O_{2}}$ [g mol^−1^]	$\rho_{Li_{2}O_{2}}$ [g m^−3^]	*h*_0_ [m]	$c_{O_{2}}$ [mol m^−3^]	*α*	*F* [C mol^−1^]	*R* [J K^−1^ mol^−1^]	*T* [K]	*k*_1_ [m s^−1^]	*k*_s_/*k*_2_
45.88	2.31 × 10^6^	15 × 10^−9^	4.43^[^ ^9^ ^]^	0.5	96 485	8.314	298	10^−10^	2.7 × 10^3^

**Figure 5:**
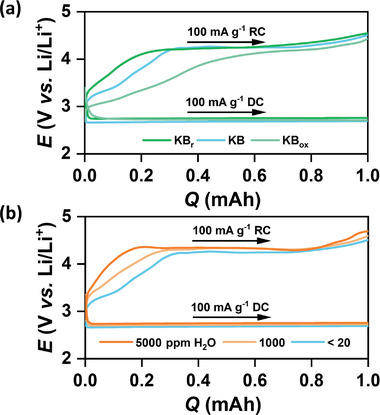
a) Potential (*E*) versus capacity (*Q*) plot of pristine and modified KB electrodes in LiTFSI/TEGDME electrolyte (<20 ppm H_2_O). b) Same plot of pristine KB electrodes in LiTFSI/TEGDME electrolyte with different water (H_2_O) content.

**Figure 6:**
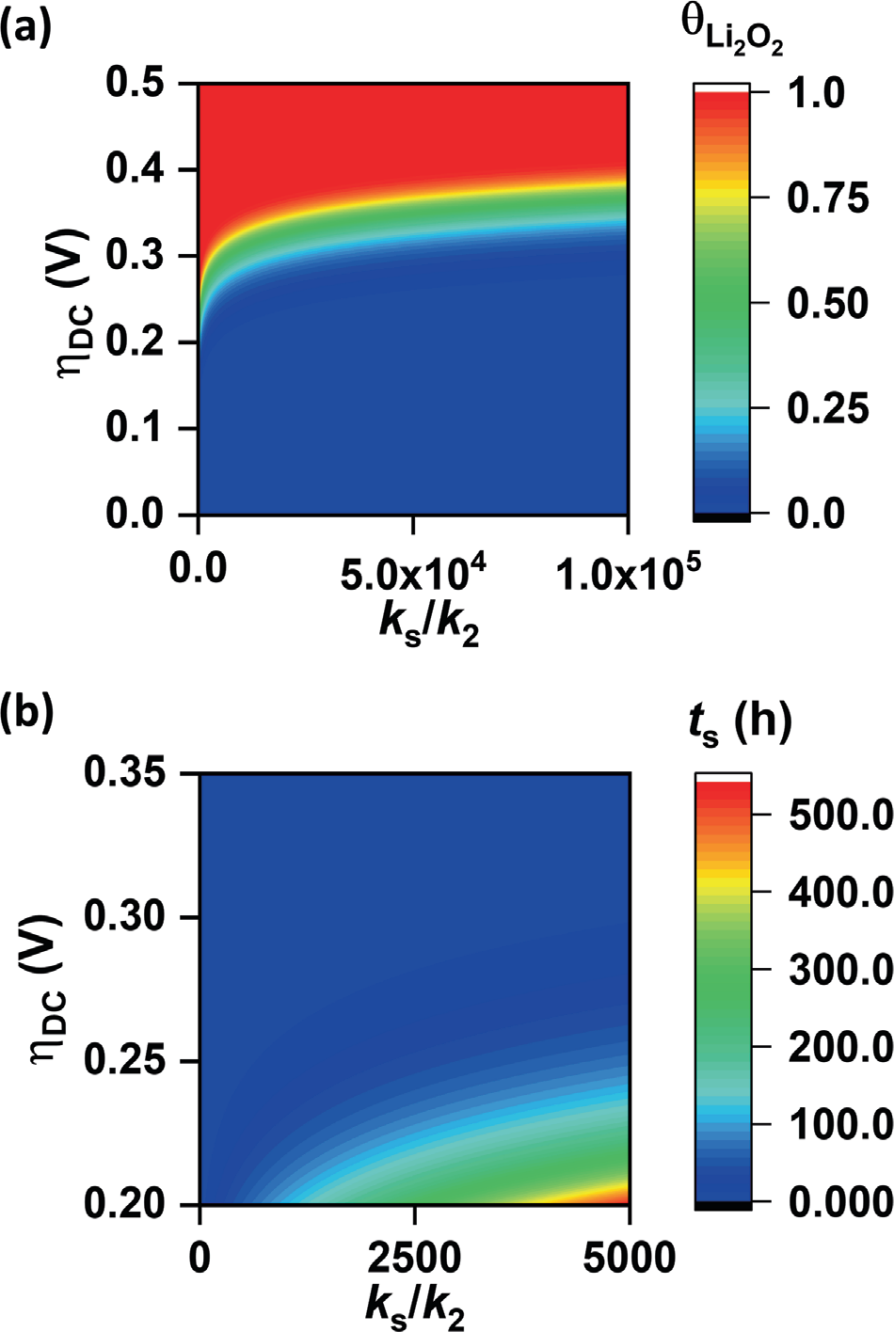
a) Contour plot showing the variation of the fraction of total electrode area covered by Li_2_O_2_ ($\theta_{Li_{2}O_{2}})$ grown by surface reduction with *η*_DC_ and the ratio of the kinetic rate constants for solution mediated disproportionation (*k*_s_) and surface reduction (*k*_2_) respectively for a fixed DC time of 10 h. b) Contour plot showing variation of *t*_s_ with *η*_DC_ and *k*_s_/*k*_2_ ratio.

$\theta_{Li_{2}O_{2}}$is the fraction of KB electrode covered by the Li_2_O_2_ layer.^[^ ^22^ ^]^ A plot of $\theta_{Li_{2}O_{2}}$ versus *j*_DC_ for *Q*_DC_ of 1 mAh and *h*_0_ value of 15 nm in Figure S12 in the Supporting Information shows gradual increase of area covered by Li_2_O_2_ film as the DC rate increases. This observation justifies the decrease in average Li_2_O_2_ film thickness with increase in *j*_DC_ as observed by TEM images in Figure 2e–h. However, we assume that, regardless of *j*_DC_, the surface grown Li_2_O_2_ layer has a fixed thickness determined by the maximum electron tunneling length through this layer. Therefore, the amount of Li_2_O_2_ deposited by surface reduction at different *j*_DC_ can be scaled with $\theta_{Li_{2}O_{2}}.\;$The actual area of the electrode covered by Li_2_O_2_ ($A_{Li_{2}O_{2}}^{\prime })\;$is calculated by multiplying ECSA of KB with $\theta_{Li_{2}O_{2}}.\;$We have estimated the amount of charge (*Q*_DC/SL_) stored in the surface grown layer in terms of deposited Li_2_O_2_ from this $A_{Li_{2}O_{2}}^{\prime }$ and different *h*_0_ values at each *j*_DC_ from 25 to 200 mA g^−1^ for a capacity of 1 mAh. Expectedly, *Q*_DC/SL_ increases with *j*_DC_ as shown in Figure 4b. We have also plotted the amount of charge passed (*Q*_RC_ < 4 V) at low RC potential (<4 V vs Li/Li^+^) during RC (both galvanostatic RC and anodic LSV) of the cells discharged up to 1 mAh at respective *j*_DC_. It is interesting to see that *Q*_DC/SL_ for a layer of 15 nm and *Q*_RC_ < 4 V measured by both galvanostatic RC and anodic LSV are quantitatively comparable. That means the 15 nm thick Li_2_O_2_ layer grown by surface reduction decomposes at potential <4 V versus Li/Li^+^. Conversely, based on this argument, any Li_2_O_2_ deposited by chemical disproportionation decomposes at higher potential (>4 V vs Li/Li^+^) flat plateau of the RC curve. This observation shows an unprecedented deconvolution of RC potentials of Li–O_2_ battery based on mechanism of Li_2_O_2_ nucleation‐and‐growth and provides a quantitative evidence that electrochemically deposited Li_2_O_2_ on the surface of the electrode decomposes at low *η*_RC_. It is important to note that this rationale does not specify the effect of any particular morphology of Li_2_O_2_ in explaining the RC curve of Li–O_2_ battery. Rather it just deconvolutes the effects of two concurrent Li_2_O_2_‐growth mechanisms on *η*_RC_. However, this general explanation can also be extended to justify the apparent effects of Li_2_O_2_ morphology on RC profile of Li–O_2_ battery. The change in morphology of Li_2_O_2_ from large toroids to conformal thin‐film is actually the shift in reaction mechanism from predominant solution phase disproportionation towards electrochemical reduction on the electrode surface. As the morphology changes from toroids to thin‐film, the fraction of surface‐grown Li_2_O_2_ increases and consequently more Li_2_O_2_ is decomposed at relatively lower *η*_RC_. Therefore, the apparent effect of Li_2_O_2_ morphology on RC potential is actually a corollary of fundamental growth mechanism described above. Although the visible morphology of Li_2_O_2_ was reported to change sharply at a particular DC condition, here we reiterate that depending on thermodynamic feasibility, the co‐occurrence of the two underlying growth processes is possible over a wide range of DC conditions and the equilibrium shifts quite steadily with small changes in external factors.^[^ ^6^, ^12^ ^]^ As a result, DC condition dependent changes in the fractions of sloping region and the high voltage plateau of a two‐stage RC profile can be simply correlated to the change in equilibrium between the Li_2_O_2_ growth mechanisms regardless of the bulk morphology. According to the nonuniform nucleation‐and‐growth mechanism explained in Figure 3, increase in depth of discharge (DOD) corresponds to progressive growth of Li_2_O_2_ in the lateral direction maintaining a fixed ratio of the two concurrent growth processes at a particular DC condition. Therefore, the ratio of the sloping region and flat plateau should not change with the DOD if the DC conditions remain unchanged. To verify this hypothesis, we have discharged and recharged three more cells up to *Q*_DC_ of 1.5, 2, and 3 mAh at *j*_DC_ of 100 mA g^−1^. The galvanostatic DC/RC curves shown in Figure S13a in the Supporting Information demonstrate similar two‐stage RC profile for all the cells. Quite interestingly, all the RC curves have overlapped upon normalization of *Q*_DC_ in Figure S13b in the Supporting Information. XRD data of the discharged electrodes shown in Figure S14a in the Supporting Information demonstrates gradual increase in Li_2_O_2_ peak intensity that signifies the addition of more Li_2_O_2_ with increase in DOD. However, no significant growth of individual grains in Li_2_O_2_ crystals is observed as shown in Figure S14b in the Supporting Information. This means that the fundamental crystallization mechanism of Li_2_O_2_ does not change with DOD and maintains same recharge efficiency. Summarizing all these results it is understood that promoting reaction (3) (surface reduction of LiO_2_*) over reaction (4) (solution phase disproportionation of LiO_2_ (sol.)) during DC can contribute to low *η*_RC_ of the cell. The competition between these two reactions represented by *k*_s_/*k*_2_ ratio depends on *η*_DC_ (or *j*_DC_) and properties of the electrode as well as of the electrolyte.^[^ ^6^, ^10^, ^17^ ^]^ We have already explained the effect of *η*_DC_ (or *j*_DC_) dependent growth process of Li_2_O_2_ on *η*_RC_. Next, in order to prove generality of our hypothesis, we have further modified KB electrode as well as the electrolyte to manipulate the *k*_s_/*k*_2_ ratio in our system. It is known that defects and surface functional groups on carbon electrode promote surface‐growth of Li_2_O_2_, whereas controlled amount of water (H_2_O) as additive in the electrolyte increases the solubility of LiO_2_ (sol.) and enhances the rate of disproportionation.^[^ ^7^, ^17^ ^]^ We have oxidized KB, hereafter denoted as KB_ox_, in H_2_SO_4_ + HNO_3_ acid mixture to introduce defects and surface oxygen functional groups that are expected to promote surface‐reduction of LiO_2_*. On the other hand, to decrease the propensity for surface reduction, KB is reduced, denoted as KB_r_, in Ar+H_2_ (4% H_2_) gas mixture at 1000 °C to remove oxygen functional groups. Raman spectra and XPS results of KB_ox_ and KB_r_ are shown in Figures S7 and S8 in the Supporting Information respectively. The *I*_D_/*I*_G_ and O/C ratios for both the samples are summarized in Table S1 in the Supporting Information. In short, these results clearly indicate significant enhancement of oxygen functionality (O/C = 0.098) and disorder (*I*_D_/*I*_G_ = 1.54) in KB_ox_ and on the contrary, after reduction at high temperature the structure has become more ordered (*I*_D_/*I*_G_ = 0.71) and oxygen content is much decreased (O/C = 0.003) in KB_r_. KB, KB_ox_, and KB_r_ are discharged and recharged at 100 mA g^−1^ up to 1 mAh and the DC/RC curves are shown in **Figure** **5**a. Unsurprisingly, as the oxygen functionality/defects increases in the electrode active material in order of KB_r_ < KB < KB_ox_, the sloping region becomes more extended with lower *η*_RC_. The span of sloping region extends from ≈25% SOC in KB_r_ to ≈33% SOC in KB to ≈60% SOC in KB_ox_. The extension of sloping region can certainly be corroborated to enhanced surface reduction of LiO_2_* with increase in surface‐functionality of the electrode. Morphologies of the as prepared and discharged KB_ox_ and KB_r_ electrodes are shown in Figure S15a–d in the Supporting Information. Figure S15b in the Supporting Information shows predominant film‐like deposition of Li_2_O_2_ on the superficial surface of KB_ox_ electrode. On the contrary, disk‐like Li_2_O_2_ with roughly 100 nm diameter and 20 nm thickness, as shown in Figure S15d in the Supporting Information, are formed on KB_r_ electrode. The large sized disk‐like particles give more evidence in favor of a dominant solution phase growth of Li_2_O_2_ in KB_r_ electrode. To further enhance the solution phase disproportionation, KB electrodes have been discharged and recharged in LiTFSI/TEGDME electrolyte with different concentrations of H_2_O added as solvating agent. Toroidal DC products in the H_2_O containing electrolytes, as shown in Figure S15e,f in the Supporting Information, certainly indicate enhanced solution mediated discharge upon addition of H_2_O. Figure 5b shows that the low‐potential sloping region becomes shorter with increase in concentration of H_2_O. In particular the length of the sloping region decreases from ≈33% SOC in dry electrolyte (<20 ppm H_2_O) to ≈27% SOC in 1000 ppm to ≈18% SOC in 5000 ppm H_2_O containing electrolytes. To provide a quantitative validation of our hypothesis that solution phase disproportionation decreases the surface coverage of Li_2_O_2_, we have calculated the dependence of $\theta_{Li_{2}O_{2}}$ on *η*_DC_ (or *j*_DC_) and *k*_s_/*k*_2_ ratio for a fixed DC time. In our calculation we have considered commonly observed *η*_DC_ values of 0–0.5 V and *k*_s_/*k*_2_ values 0–10^5^ at a DC time of 10 h. Here *k*_s_/*k*_2_ = 0 implies exclusive surface reduction of LiO_2_*, whereas *k*_s_/*k*_2_ = 10^5^ means greater contribution from solution phase disproportionation of LiO_2_ (sol.). The contour plot in **Figure** **6**a shows that at a particular *η*_DC_, $\theta_{Li_{2}O_{2}}$ increases with decrease in *k*_s_/*k*_2_ value. On the other hand, increase in *η*_DC_ that enhances the rate of LiO_2_* reduction (reaction (3)) also increases $\theta_{Li_{2}O_{2}}$ at a fixed value of *k*_s_/*k*_2_. This clearly demonstrates that with the enhancement of LiO_2_* reduction, more fraction of electrode surface gets covered with Li_2_O_2_. Although increase in $\theta_{Li_{2}O_{2}}$ decreases the *η*_RC_ as explained above, at the same time the electrode surface becomes passivated with insulating layer of Li_2_O_2_ that causes early death of DC resulting in capacity‐rechargeability trade‐off in Li–O_2_ battery. In Figure 6b we have plotted the sudden‐death time (*t*_s_) at different *η*_DC_ and *k*_s_/*k*_2_ values. The results unequivocally demonstrate that dominant surface reduction of LiO_2_* leads to early death and hence manifests low *Q*_DC_.

### 2.5 Establishing Mechanism of Low‐Potential Recharge Pathway

**Figure 7:**
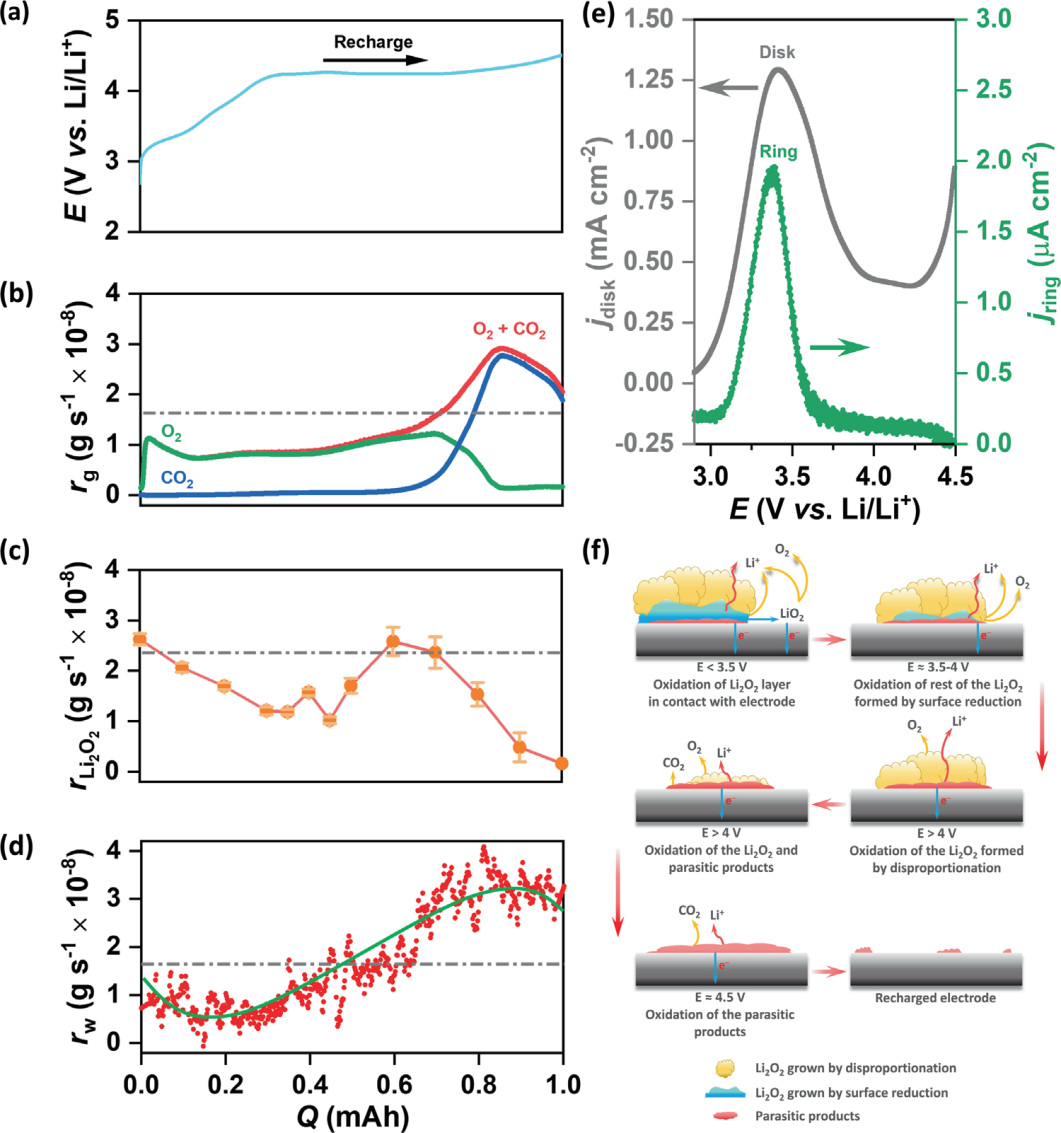
a) *E* versus *Q* plot for RC of KB electrode at *j*_DC_ of 100 mA g^−1^ in LiTFSI/TEGDME electrolyte (<20 ppm H_2_O). b) Online electrochemical mass spectrometry (OEMS) results showing the rate of gas evolution (*r*_g_), c) Rate of decomposition of Li_2_O_2_ ($r_{Li_{2}O_{2}})$ measured by titration of the electrodes with TiOSO_4_ solution at different SOCs and d) Rate of mass change of the whole cell by in situ weight measurement during RC of the cells. e) Anodic linear sweep voltammetry (LSV) plot using rotating ring disk electrode (RRDE) with KB disk and glassy carbon (GC) ring at a sweep rate of 5 mV s^−1^ and rotation speed of 900 rpm. f) Schematic representation of the proposed RC mechanism. The broken gray lines in b)‐d) represent the rates corresponding to 2e^−^/O_2_ process. The green line in (d) is just a visual guide to the trend of the data.

In order for developing a more efficient Li–O_2_ battery, it is necessary to understand why and how the surface‐grown Li_2_O_2_ decomposes at relatively lower *η*_RC_. Therefore, we now focus on understanding and establishing the RC mechanisms that correlate to the decompositions of surface and solution‐grown Li_2_O_2_ at different RC potentials. The apparent sharp transition from a sloping trend to a flat plateau in the RC curve (Figure 1b) and two markedly different oxidation peaks (≈3.35 and >4.2 V vs Li/Li^+^) in anodic LSV (Figure 1c; Figure S2, Supporting Information) certainly indicate a switch in decomposition mechanism for these two types of Li_2_O_2_. Since RC of Li–O_2_ battery involves oxidation of ostensibly insulating solid Li_2_O_2_, *η*_RC_ seems to be sensitive towards subtle structural differences that affect charge transport through the solid Li_2_O_2_ layer.^[^ ^15^ ^]^ As it has been mentioned earlier in this article, due to higher adsorption energies of lithium and oxygen, Li_2_O_2_ starts nucleating preferentially at the defect sites on carbon surface.^[^ ^19^, ^23^ ^]^ According to prior DFT calculations, the strong binding interactions between nucleating sites on the carbon surface and Li_2_O_2_ lead to electron transfer from the anti‐bonding O_2_ orbitals to the carbon electrode inducing a p‐type conductivity at Li_2_O_2_/electrode interface.^[^ ^24^ ^]^ Furthermore, due to this strong binding, reorganization of bonding electrons often results in elongation of Li–O bond length that diminishes the interaction between lithium atom and oxygen atom in Li_2_O_2_.^[^ ^25^ ^]^ As a result, mobility of Li^+^ in Li_2_O_2_ crystal becomes easier in the layer that interacts strongly with carbon electrode surface. These defect‐induced improvements in both ionic and electronic conductivities of surface grown Li_2_O_2_ perhaps make the decomposition possible at low *η*_RC_. In contrast, the Li_2_O_2_ precipitated from solution phase is believed to be structurally more ordered, stoichiometric and electronically insulating that requires high *η*_RC_ for decomposition.^[^ ^7^, ^15^ ^]^ The higher structural order of solution grown Li_2_O_2_ is evinced by the XRD patterns shown in Figure S16a,b in the Supporting Information. The XRD data in Figure S16a in the Supporting Information show that the intensity of peaks and the average domain size of Li_2_O_2_ crystals increases as the propensity towards solution growth increases on different carbons in the order KB_ox_ < KB < KB_r_. Consistent with this trend, Figure S16b in the Supporting Information shows, increasing H_2_O content in the electrolyte also increases both the XRD peak intensity and the domain size in Li_2_O_2_ crystals. **Figure** **7**a–d shows galvanostatic RC curve in combination with quantitative estimation of OER to gain clear understanding of the RC process. The online electrochemical mass spectrometry (OEMS) data in Figure 7b show a plot of gas evolution rate (*r*_g_) with *Q*_RC_. A high rate ($r_{g/O_{2}}$) of oxygen evolution at the beginning of RC confirms the decomposition of Li_2_O_2_ below 3.5 V versus Li/Li^+^. However, the $r_{g/O_{2}}$ value is lower than that calculated for a 2e^−^/O_2_ process and more surprisingly, although the RC potential gradually increases, $r_{g/O_{2}}$ shows a decreasing trend up to ≈15% SOC that corresponds to ≈3.5 V versus Li/Li^+^. This means either the rate of Li_2_O_2_ decomposition ($r_{Li_{2}O_{2}})$ is lower than expected with a similar decreasing trend or the evolved oxygen somehow remains trapped inside the electrode during the course of RC. In order to quantify $r_{Li_{2}O_{2}}$, we have titrated the KB electrode at different SOCs by TiOSO_4_ solution and the results are shown in Figure 7c. The titration data show that the $r_{Li_{2}O_{2}}$ values are quite high, roughly close to the theoretical value of 2.38 × 10^−8^ g s^−1^, at the beginning of RC and remain significantly higher than $r_{g/O_{2}}$ up to ≈30% SOC. These results evince that the decomposition of Li_2_O_2_ is considerably facile at low *η*_RC_ sloping region. The difference between $r_{g/O_{2}}$ and $r_{Li_{2}O_{2}}$ can be attributed to generation of highly reactive singlet oxygen (^1^O_2_) that reacts with electrode as well as with electrolyte and does not evolve to be detected by OEMS despite continuous decomposition of Li_2_O_2_ during RC.^[^ ^26^ ^]^ These oxidations of electrode and electrolyte by ^1^O_2_ produce parasitic side products in the form of Li_2_CO_3_, RCO_2_Li, etc.^[^ ^16a^ ^]^ We have also measured the change in mass of the whole cell in situ during DC/RC. Ideally, any change in the mass of a cell should account for the consumption and evolution of oxygen during DC and RC respectively. We have estimated a ≈1.97 e^−^/O_2_ ORR from the mass of consumed O_2_ during DC of the cell and the trend of O_2_ consumption is shown in Figure S17 in the Supporting Information. On the other side Figure 7d shows the rate of mass change (*r*_w_) of the cell during RC. Generally, the value of *r*_w_ should account for the decrease in mass due to oxygen evolution from decomposition of Li_2_O_2_. Interestingly, despite high value of $r_{Li_{2}O_{2}}$ the graph in Figure 7d shows a decreasing trend of *r*_w_ up to ≈30% SOC and follows the trend of oxygen evolution. This observation clearly indicates entrapment of oxygen and accumulation of insulating side products that balance the decrease in mass due to Li_2_O_2_ decomposition. Nevertheless, the non‐catalytic decomposition of Li_2_O_2_ below 3.5 V versus Li/Li^+^ provides optimism about achieving Li–O_2_ batteries with high round‐trip energy efficiency. Considering the high energy barrier for a direct 2e^−^ oxidation, stepwise 1e^−^ process with low delithiation barrier seems to be a preferred oxidation pathway for Li_2_O_2_.^[^ ^8c^ ^]^ Advantageously, defect induced structural disordering of surface grown Li_2_O_2_ lowers both the formation and migration energies of Li^+^ vacancy leading to facile delithiation and formation of non‐stoichiometric Li_2−_ *_x_*O_2_ (0 < *x* < 1) phase.^[^ ^27^ ^]^ Despite the energetically favorable delithiation, according to first‐principles calculations, oxygen evolution from the solid Li_2−_ *_x_*O_2_ species remains as the highest energy‐demanding step during Li_2_O_2_ decomposition.^[^ ^28^ ^]^ Alternatively, a concerted delithiation of Li_2_O_2_ clusters leading to disintegration and dissolution of the non‐stoichiometric Li_2−_ *_x_*O_2_ at a critical value of *x* may instead provide a solution‐phase oxidation pathway on the bare electrode surface at relatively low *η*_RC_ in the range 0.3–0.4 V.^[^ ^27^ ^]^ An anodic LSV has been carried out on a pre‐discharged KB electrode using RRDE set up similar to that described for Figure S6 in the Supporting Information. The current responses of the KB disk swept from 2.9 to 4.5 V versus Li/Li^+^ and the GC ring held at 3.5 V versus Li/Li^+^ are shown in Figure 7e. It is indeed found that the oxidation peak for the surface grown Li_2_O_2_ at ≈3.4 V versus Li/Li^+^ is associated with the occurrence of ring current at the GC ring electrode. This observed ring current directly provides evidence in favor of a partial solution mediated oxidation of the Li_2_O_2_ that is deposited by surface reduction. The oxidation in the solution phase is likely to be energetically favorable over the solid phase oxidation due to circumvention of the charge transport limitation of insulating solid Li_2_O_2_. Interestingly, the rationale for decomposition of surface‐grown Li_2_O_2_ at low *η*_RC_ can be extended to explain the apparent electro‐catalytic effect in Li–O_2_ battery. Any catalyst that shows strong binding towards Li^+^ and O_2_ is found to promote surface‐growth of Li_2_O_2_ and subsequently exhibits low *η*_RC_.^[^ ^25^, ^29^ ^]^ Therefore, regardless of the electrode type, low *η*_RC_ is fundamentally linked to the Li_2_O_2_ growth on the surface of the electrode during DC. Now the question is why the potential gradually increases and forms a slope before reaching a flat plateau. One possible reason is the structural nonuniformity in the surface‐grown Li_2_O_2_ layers that may show a range of RC potential. According to DFT calculations, the interaction between carbon and the Li_2_O_2_ layers changes as the distance from the carbon surface increases.^[^ ^24^ ^]^ As a result, the degrees of disorder in the surface‐grown Li_2_O_2_ is also expected to change and may exhibit gradual change in RC potential. Another obvious reason for increase in RC potential is the accumulation of parasitic side products that increase the impedance of the cell.^[^ ^16^ ^]^ After complete decomposition of surface grown Li_2_O_2_ when the RC potential reaches the plateau and *η*_RC_ becomes high (>1 V), the solution grown structurally more ordered and stoichiometric Li_2_O_2_ starts decomposing. Based on first‐principles calculations, the insulating bulk Li_2_O_2_ becomes conductive only when the *η*_RC_ is high enough to activate the delithiation process and the charge transport arises from the mixture of Li vacancies and hole polaron's contributions.^[^ ^11^ ^]^ Therefore, oxidation of Li_2_O_2_ in this case also presumably starts with delithiation and progresses via non‐stoichiometric phases. In fact, decomposition of Li_2_O_2_ by forming Li vacancy in the crystal was indeed observed by an in situ XRD study during RC of a Li–O_2_ battery.^[^ ^14a^ ^]^ However, unlike decomposition of surface grown Li_2_O_2_, no solution mediated oxidation has been observed for the Li_2_O_2_ grown by disproportionation. High enough *η*_RC_ to permit successive delithiation and oxygen evolution from the Li_2_O_2_ crystal before it disintegrates and dissolves in the electrolyte may be the reason why no solution mediated oxidation is observed in this case. It is interesting that although the galvanostatic RC curve reaches a flat‐potential (≈4.2 V vs Li/Li^+^) plateau at ≈30% SOC and roughly maintains the potential up to ≈80% SOC, the values of $r_{g/O_{2}}$ and $r_{Li_{2}O_{2}}$ are not consistent with this Coulometric data. Both $r_{g/O_{2}}$ and $r_{Li_{2}O_{2}}$ are quite low at the beginning of the plateau and steadily increases up to 60–70% SOC where in fact $r_{Li_{2}O_{2}}$ reaches the theoretical value. These low values of $r_{g/O_{2}}$ and $r_{Li_{2}O_{2}}$ can be attributed to delithiation or Li vacancy creation process in stoichiometric and structurally more ordered solution grown Li_2_O_2_ at the initial part of the plateau.^[^ ^6b^ ^]^ This is consistent with the oxygen evolution trend during RC of solution grown large toroidal Li_2_O_2_ that also shows very low $r_{g/O_{2}}$ at ≈5–20% SOC as demonstrated in Figure S18 in the Supporting Information. After this initial delithiation process, formation of non‐stoichiometric phases and gradual decrease in both particle size and thickness of Li_2_O_2_ deposits leading to improved charge transport can be one of the reasons behind the high OER rate at the later stage of RC. A possible mechanism for the decomposition of Li_2_O_2_ is schematically described in Figure 7f. After complete decomposition of Li_2_O_2_, high amount of carbon dioxide (CO_2_) is evolved due to decomposition of parasitic products. From the ratio of consumed oxygen to evolved oxygen measured by in situ mass measurement and OEMS respectively, we have calculated an OER/ORR ratio of 0.47. Considering ≈70% yield of Li_2_O_2_ after DC, a correction of OER/ORR ratio gives a value of 0.67 which still indicates loss of oxygen during RC due to parasitic reactions. As a result, the overall mass change rate (*r*_w_) of the electrode does not follow the trend of $r_{Li_{2}O_{2}}$ rather shows excellent consistency with the cumulative rate of gas evolution (*r*_g_shown by the red curve in Figure 7b) that include the information about parasitic reactions. The problem of parasitic reactions also affects the cycling stability of the electrodes. Figure S19a–c in the Supporting Information shows the 10 galvanostatic cycles of KB, KB_r_ and KB_ox_ electrodes, respectively. Deterioration of the cycling performance is evident in all the cases. However, the comparison of the 5th and 10th cycles of these electrodes in Figure S20a,b in the Supporting Information, respectively, shows that KB_ox_ consistently maintains the lowest RC potential among these three electrodes. Nonetheless, considering gradual increase in *η*_RC_ over the cycles, it should be noted that in order to achieve an efficient Li–O_2_ battery with large capacity, high rechargeability and long cycle life, it is essential to improve the reversibility of ORR and OER by mitigating the problem of parasitic reactions.

## 3 Conclusion

In summary, we have investigated the recharge mechanism of Li–O_2_ battery in order to identify a non‐catalytic low energy decomposition route for Li_2_O_2_. The onset potential for Li_2_O_2_ decomposition has been found to be very close to the equilibrium potential of 2.96 V versus Li/Li^+^. However, during the course of recharge, the potential gradually increases, goes above 4 V versus Li/Li^+^ and forms a flat‐potential plateau. Our detailed analyses have shown that neither the bulk morphology of Li_2_O_2_ nor the parasitic side reactions can completely explain this two‐stage recharge profile. Here we have demonstrated that the recharge potential of a Li–O_2_ battery can rather be fundamentally correlated to the crystallization pathway of Li_2_O_2_. Depending on thermodynamic feasibility, Li_2_O_2_ crystallization occurs by concurrent electrochemical surface reduction and solution mediated chemical disproportionation reactions over a wide range of discharge conditions. A combination of experimental data and theoretical calculations shows, the Li_2_O_2_ grown by these two routes forms two coexisting discrete phases with subtle structural differences. In an unprecedented attempt, we have deconvoluted and quantitatively estimated the amount of Li_2_O_2_ deposited by these two different processes. Our quantitative analyses precisely specify that the surface‐grown structurally disordered Li_2_O_2_ decomposes at relatively low recharge potential (<3.5 V vs Li/Li^+^). Detailed mechanistic study identifies a delithiation induced partial solution phase decomposition of surface grown Li_2_O_2_ to be a non‐catalytic low energy oxidation pathway. On the contrary, regardless of bulk morphology, solution‐grown Li_2_O_2_ decomposes at recharge potential >4 V versus Li/Li^+^. Therefore, any discharge condition that promotes predominant surface growth of Li_2_O_2_ should be beneficial for efficient recharge of the cell. We have further shown that the equilibrium between these two growth processes and the subsequent recharge profile can be controlled by the properties of the electrode as well as of the electrolyte. In short, our results provide important insights into necessary control over fundamental crystallization pathways of Li_2_O_2_ by proper choice of electrode and electrolyte to improve the energy efficiency of Li–O_2_ battery.

## Conflict of Interest

The authors declare no conflict of interest.

## Supporting information

Supporting Information
