# Supplementary material for: Quantitative Delineation of the Low Energy Decomposition Pathway for Lithium Peroxide in Lithium–Oxygen Battery
Source: Adv Sci (Weinh). 2020 Aug 11;7(19):2001660. doi: 10.1002/advs.202001660 (PMC7539218; doi:10.1002/advs.202001660)
Supplement: Supplementary file 1 — Supporting Information [file ADVS-7-2001660-s001.pdf]

© 2020 Wiley-VCH GmbH

ADVANCED SCIENCE
Open Access

Supporting Information

for *Adv. Sci.*, DOI: 10.1002/advs.202001660

Quantitative Delineation of the Low Energy Decomposition Pathway for Lithium Peroxide in Lithium–Oxygen Battery

Arghya Dutta, Kimihiko Ito, Akihiro Nomura, and Yoshimi Kubo**

© 2020 Wiley-VCH GmbH

Supporting Information

Quantitative Delineation of the Low Energy Decomposition Pathway for Lithium Peroxide in Lithium-Oxygen Battery

Arghya Dutta*, Kimihiko Ito, Akihiro Nomura and Yoshimi Kubo*

1. Experimental section

1.1. Electrode preparation: Ketjenblack (KB) (EC600JD, Lion) particles, having ~ 40-60 nm diameter, were used as the positive electrode materials. Oxidation of KB was carried out by adding KB (500 mg) to a 3:1 mixture of concentrated H_2SO_4 and HNO_3 (10 mL) and was stirred for 2 h at 70 ℃. After cooling down, the solution was centrifuged, washed with H_2O until no acid in the washing liquid was detected by pH paper and dried at 110 ℃ under vacuum. Oxygen functional groups in KB were reduced by heating pristine KB at 1000 ℃ for 5 h under continuous flow of argon (Ar) gas containing 4% hydrogen (H_2). Slurries of different carbons were prepared by mixing with LITHion™ (Ion Power) binder (4:1 ratio (w/w) for carbon/LITHion) in N-methyl-2-pyrrolidone (NMP) solvent and the slurries were tape casted on carbon paper (CP, TGP-H-30, Toray). The electrodes were dried slowly at room temperature followed by vacuum drying at 110 ℃ for 12 h, and finally cut into 1.6 cm diameter electrode (2 cm^2 geometric area). The average carbon loading for all the electrodes was ~ 0.5 mg cm^{-2}.

1.2. Li–O_2 cell assembly and testing: All the Li–O_2 cells were assembled and tested inside a dry-room (dew point of < -60 ℃) and were never exposed to ambient conditions. Coin type CR2032 cells with perforated stainless-steel cases (Hohsen Corp.) were used and the cells were assembled inside an isolated draft chamber under oxygen atmosphere with dew point of < -90 ℃ (< 0.1 ppm H_2O). The Li–O_2 cells were made with a Li metal (1.6 cm in diameter, 200 μm thickness, Honjo Metal) negative electrode, two separators of Celgard® 2325 (1.6 cm in diameter) and glass fiber (GF/C, 1.6 cm in diameter, Whatman®) and the positive electrode of different carbon materials. 1 M Lithium bis(trifluoromethanesulfonyl)imide (LiTFSI, Kishida Chemical Co., Ltd.) dissolved in anhydrous (< 20 ppm H_2O measured by Karl Fisher titration) and water-added (1000 and 5000 ppm H_2O) tetraethylene glycol dimethyl ether (TEGDME, Japan Advanced Chemicals) were used as the non-aqueous electrolytes (100 μL). The Li–O_2 coin cells were set inside a closed box connected to oxygen gas line and cell testing was carried out under flowing oxygen (dew point of < -90 °C (< 0.1 ppm H_2O)) using a galvanostatic electrochemical tester (HJ1001SD8, Hokuto Denko) at ~ 25 °C. Linear sweep voltammetry (LSV) of the coin cells was performed with a Biologic (VSP-300) potentiostat/galvanostat.

Inside the cell-testing box, oxygen gas can enter the cell through the perforated stainless-steel case and diffuse into the electrode as described in Figure 1 (a). Cyclic voltammetry (CV) experiment of symmetric KB|separator|KB coin cell to measure electrochemically active surface area (ECSA) was tested on an Autolab PGSTAT128N in the range 0 to 1 V at a sweep rate of 50 mV s^{-1}. Linear sweep voltammetry (LSV) measurements under 3-electrode set up were carried out using Autolab PGSTAT128N and a Pine Modulated Speed Rotator (MSR) fitted with rotating ring disk electrode (RRDE) where KB was used as the disk and glassy carbon (GC) was the ring electrode, while metallic lithium served as both counter and reference electrodes in 1 M LiTFSI/TEGDME electrolyte. KB slurry, similar to mentioned above, was drop-casted on a GC disk (0.25 cm^2) to prepare the electrode. The sweep rate and rotation speed were 5 mV s^{-1} and 900 rpm respectively. During the potential sweep at the disk, the ring potential was held at 3.5 V *vs*. Li/Li^+. Oxygen gas was bubbled through the electrolyte for 1 h before LSV to ensure saturation and oxygen was continued to flow through the vessel at the time of measurement.

1.3. Titration of discharged and recharged electrodes with titanium (IV) oxysulfate ($TiOSO_4$) solution: Cells discharged and recharged up to different capacities were disassembled and the electrodes along with separators were soaked in $TiOSO_4$ solution (3 mL) (1.9-2.1%, Sigma Aldrich) + Milli-Q® H_2O (10 mL) for 1 h. The absorbances of the solutions obtained by soaking different electrodes were measured by a Shimadzu UV 2600 UV-vis spectrophotometer at the wavelength 407 nm. Quantification of Li_2O_2 in the electrodes were done by correlating the measured absorbance with calibration curve reported elsewhere.[1] In short, for calibration, absorbances of several known amounts of commercial Li_2O_2 (> 90%, Sigma Aldrich) (weighed by Cubis® Micro Balance (Sartorius)) dissolved in same volume of $TiOSO_4$ and H_2O as stated above, were measured and plotted against Li_2O_2 mass.

1.4. Electrochemically active surface area (ECSA) measurement:[1] ECSA of the KB electrode was measured by cyclic voltammetry (CV) of symmetric KB|separator|KB coin type 2032 cells with 100 μL 1 M LiTFSI/TEGDME electrolyte (< 20 ppm H_2O) in the range 0 to 1 V at a sweep rate of 50 mV s^{-1}. At first, the capacitance of the KB electrode was calculated by using the equation

$$C = \int_{E_1}^{E_2} \frac{i\,(E)dE}{2(E_2\text{-}E_1)mv}$$

where C is the specific capacitance of a single KB electrode. E_1, E_2 are the lower and upper limits of cut-off potentials for CV respectively, i (E) is the instantaneous current, m is the mass

of a single KB electrode and v is the scan rate of CV. The capacitance was calculated from 400th cycle of CV.

After obtaining the specific capacitance, ECSA of an electrode was calculated from:

$$C = \frac{\varepsilon\, \varepsilon_0\, A}{d}$$

where ε is the relative dielectric constant of teraglyme (7.79), ε_0 is the permittivity of vacuum (8.85×10^{-12} F m^{-1}), A is the ECSA and d is the electric double layer thickness assumed to be the radius of oxygen molecules (~1.5 Å).

The ECSA of KB electrode was calculated to be 28 m^2 g^{-1} that equates to 0.028 m^2 for a single electrode having mass of 1 mg. This measured ECSA value of KB electrode is quite consistent with previous report.[2] Expectedly, the ECSA of KB electrode is much smaller than the Brunauer-Emmett-Teller (BET) surface area of the KB powder. The BET technique measures the total specific surface area of a material by physical adsorption of nitrogen (N_2) at -196 ℃ whereas, ECSA measured by CV represents the actual electrode area accessible to liquid electrolyte and active for electrochemical reactions.

1.5. Characterization: After discharge, the cells were disassembled, electrodes were rinsed with dehydrated tetrahydrofuran (< 10 ppm H_2O, Wako), dried under vacuum and used for post-mortem analyses. Hermetic transfer vessels were used in all cases to carry samples and no sample was exposed to ambient atmosphere during characterization. Powder X-ray diffraction (XRD) patterns of the discharged electrodes were collected on a D8 ADVANCE (Bruker) diffractometer with a CuKα source (λ CuKα = 1.542 Å). Fourier transform infrared (FTIR) spectra were obtained using Nicolet iS50 (Thermo Fisher Scientific) in Attenuated total reflection (ATR) mode with DLaTGS-CsI detector and CsI beamsplitter. X-ray photoelectron spectra (XPS) were collected using a VersaProbe II Scanning XPS Microprobe (ULVAC-PHY). Raman spectra of KB and modified KB samples were obtained in Nanophoton RAMANtouch-VIS-NIR. Morphologies of the discharged products were observed on a JSM-7800F field-emission scanning electron microscope (FE-SEM, JEOL) with 5 keV accelerating voltage and JEOL JEM-ARM200F (accelerating voltage 200 kV) transmission electron microscope (TEM). UV-vis spectra were collected on a Shimadzu UV 2600 spectrophotometer. Online electrochemical mass spectrometry (OEMS) measurements were carried out in a home-made system using a quadrupole mass spectrometer (JMS-Q1500, JEOL). The electrochemical flow cells for OEMS were assembled using the same components (Li|separator|carbon) as described above. The gas evolution during charging was continuously measured by flowing He as the carrier gas at a rate of 2 mL min^{-1}. The weight of a coin cell (CR2032) during the

discharge/charge process was measured by an automatic electronic balance (AD-4212B-23, A&D, Japan). The balance weighed the cell automatically every minute with performing zero correction each time, which enabled long-term monitoring of the cell weight with the accuracy of the balance used. The profile of cell weight change was obtained by subtracting the initial cell-weight from the chronological amount of cell weights and a correction for electrolyte evaporation was carried out by continuously measuring the weight of a cell kept at rest for 48 h.

1.6. Statistics: All data are presented as mean ± standard deviation from at least three independent experiments, except OEMS and in situ weight measurements. All the XRD data were normalized to the intensity of the KB+CP peak at 2θ = 54.3. Wide scan XPS were normalized to the C1s peak at ~ 284 eV. All graphical plots and curve fitting were carried out using OriginPro 2019b software (OriginLab Corporation, Northampton, MA, USA).

2. Theory

2.1. Derivation of areal growth rate of Li_2O_2: We assume Li_2O_2 is formed on the electrode surface by concurrent nucleation-and-growth. 'Nucleation' refers to progressive formation of Li_2O_2 at the active sites by surface reduction and grows horizontally along the surface maintaining a fixed film height. On the other hand, 'growth' refers to deposition of Li_2O_2 on surface-grown Li_2O_2 by solution phase disproportionation and grows vertically.

The rate of oxygen reduction (r_1) forming LiO_2 and subsequent reduction of surface adsorbed LiO_2* (r_2)can be represented by

$$r_1 = k_1 c_{O_2} \exp(-\frac{\alpha_1 F}{RT}\eta_1) \quad \text{(S1)}$$

$$r_2 = k_2 c_{LiO_2} \exp(-\frac{\alpha_2 F}{RT}\eta_2) \quad \text{(S2)}$$

Where k_1 (m s^{-1}) and k_2 (s^{-1}) are the rate constants for reduction of surface adsorbed oxygen (O_2*) and surface adsorbed lithium superoxide (LiO_2*) respectively, c_{O_2} (mol m^{-3}) and c_{LiO_2} (mol m^{-2}) are the concentrations of oxygen in the electrolyte and surface concentration of LiO_2* on the electrode respectively, α_1 and α_2 are the charge transfer symmetry factors approximated to be 0.5, η_1 and η_2 are the overpotentials for the respective steps assumed to be $\eta_1 \approx \eta_2 = \eta_{DC}$. F (96485 C mol^{-1}), R (8.314 J mol^{-1} K^{-1}) and T (298 K) have their usual meaning. The solvation kinetics of LiO_2* is represented by

$$r_s = k_s c_{LiO_2} \quad \text{(S3)}$$

Applying mass balance and steady state conditions for LiO_2*

$$\frac{d}{dt}c_{LiO_2} = r_1 - r_2 - r_s = 0$$

$$\frac{r_1}{r_2} = 1 + \frac{r_s}{r_2} \tag{S4}$$

From equations (S1) and (S2),

$$c_{LiO_2} = \frac{r_2}{r_1} \times \frac{k_1}{k_2} \times c_{O_2}$$

Substituting the value of r_2/r_1 from equation (S4)

$$c_{LiO_2} = \frac{1}{(1+\frac{r_s}{r_2})} \times \frac{k_1}{k_2} \times c_{O_2}$$

Putting values of r_2 and r_s from equations (S2) and (S3)

$$c_{LiO_2} = \frac{1}{(1+\frac{k_s}{k_2 \exp(-\frac{\alpha F}{RT}\eta_{DC})})} \times \frac{k_1}{k_2} \times c_{O_2}$$

$$c_{LiO_2} = \frac{k_1 \times \exp(-\frac{\alpha F}{RT}\eta_{DC})}{k_2 \times \exp(-\frac{\alpha F}{RT}\eta_{DC}) + k_s} \times c_{O_2} \tag{S5}$$

Charge and mass balance for Li_2O_2 gives the rate of volume change

$$\frac{dV_{Li_2O_2}}{dt} = (\frac{M_{Li_2O_2}}{F \times \rho_{Li_2O_2}} \times I_2) + S$$

where $M_{Li_2O_2}$ and $\rho_{Li_2O_2}$ are the molar mass (45.88 g mol^{-1}) and bulk density (2.31 × 10^6 g m^{-3}) of Li_2O_2 respectively. Here the term in the parenthesis accounts for the volume change due to reduction of LiO_2* and the term S represents contribution from chemical disproportionation of LiO_2 (sol.) from solution. According to our assumption, the areal growth depends on the surface reduction of LiO_2*. Therefore, we consider only the amount of Li_2O_2 produced by electrochemical reduction to calculate the areal growth rate. We further assume that the Li_2O_2 layer on the surface has a fixed average thickness (h_0). Based on these assumptions, the areal growth rate is

$$\frac{dA_{Li_2O_2}}{dt} = A_{electrode} \times \frac{M_{Li_2O_2}}{\rho_{Li_2O_2} \times h_0} \times k_2 \times c_{LiO_2} \times \exp(-\frac{\alpha F}{RT}\,\eta_{DC})$$

Putting value of c_{LiO_2} from equation (S5)

$$\frac{dA_{Li_2O_2}}{dt} = A_{electrode} \times \frac{M_{Li_2O_2}}{\rho_{Li_2O_2} \times h_0} \times c_{O_2} \times \frac{k_1 \times \exp(-\frac{2\alpha F}{RT}\eta_{DC})}{\exp\left(-\frac{\alpha F}{RT}\eta_{DC}\right) + \frac{k_s}{k_2}} \tag{S6}$$

End of discharge is defined by full coverage of Li_2O_2 film on electrode surface:

$$A_{Li_2O_2} = A_{electrode}.$$

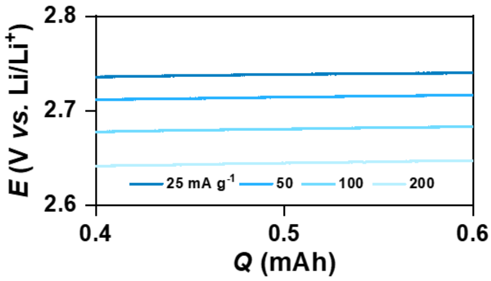

Figure S1. A magnified view of the discharge curves showing potential (*E*) *vs*. capacity (*Q*) plots from Figure 1 (b) in the range 0.4-0.6 mAh.

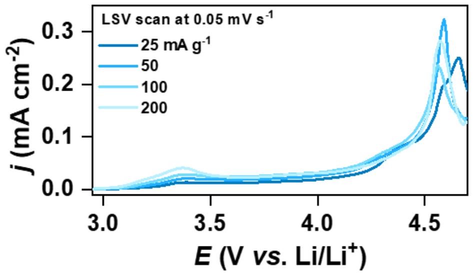

Figure S2. Anodic linear sweep voltammetric (LSV) curves of KB electrodes in the range 2.95-4.7 V *vs*. Li/Li^+ after discharge at different j_{DC} up to the fixed capacity (Q_{DC}) of 1 mAh.

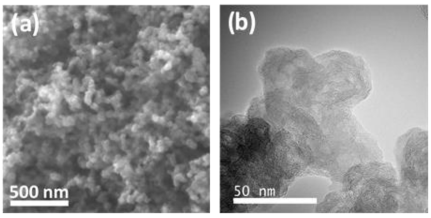

Figure S3. (a) Scanning electron microscopy (SEM) and (b) transmission electron microscopy (TEM) images of pristine KB sample.

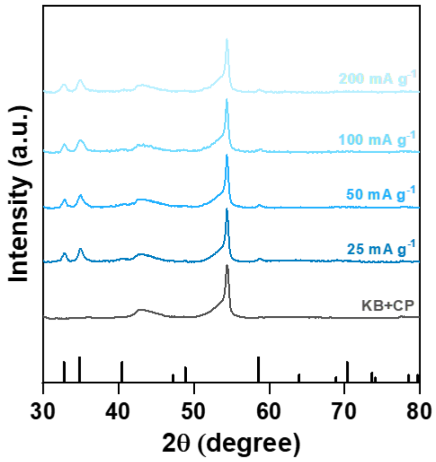

Figure S4. X-ray diffraction (XRD) patterns of the KB electrodes after discharge in LiTFSI/TEGDME electrolyte (< 20 ppm H_2O) at different j_{DC} up to a Q_{DC} of 1 mAh. All the XRD data are normalized to the intensity of the KB+CP peak at 2θ = 54.3. Peak positions for standard Li_2O_2 sample are shown by black vertical lines at the bottom of the plot. XRD of as-prepared KB+CP electrode is also shown for comparison.

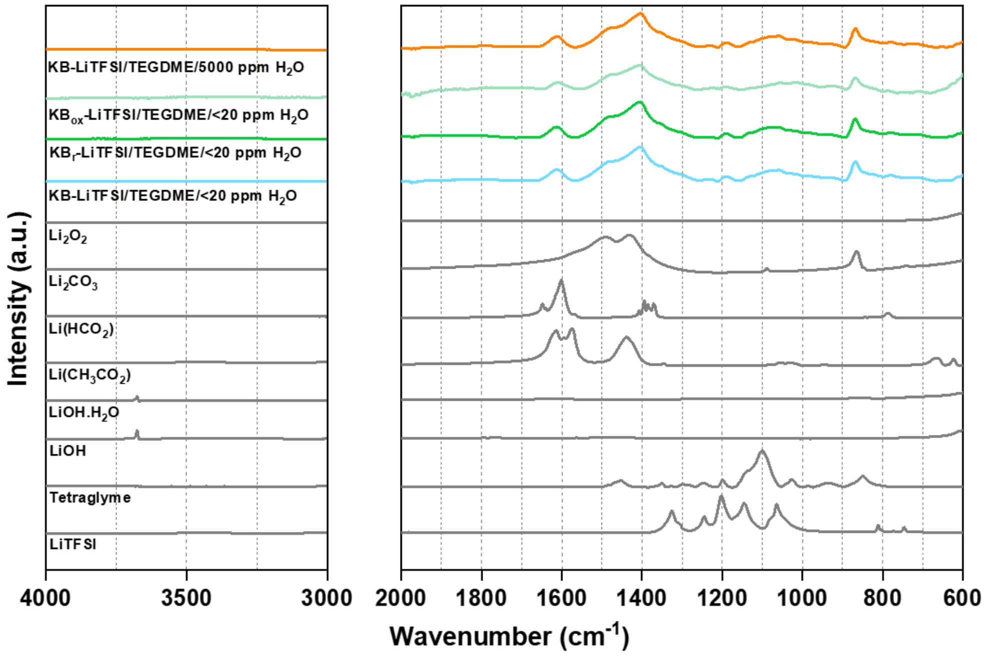

Figure S5. Fourier transform infrared (FTIR) spectra of the pristine and modified KB electrodes after discharge up to a Q_{DC} of 1 mAh at j_{DC} of 100 mA g^{-1} in < 20 and 5000 ppm H_2O containing LiTFSI/TEGDME electrolyte. Spectra of several lithium salts as potential side products are also shown for comparison.

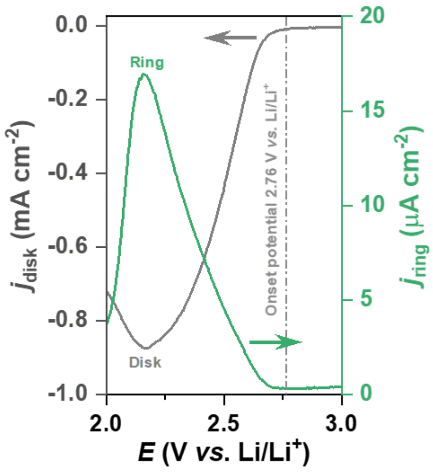

Figure S6. Cathodic linear sweep voltammetry (LSV) plot using rotating ring disk electrode (RRDE) with KB disk and glassy carbon (GC) ring at a sweep rate of 5 mV s^{-1} and rotation speed of 900 rpm. KB disk electrode is swept from 3 to 2 V *vs*. Li/Li^+ while the glassy carbon ring is held at 3.5 V *vs*. Li/Li^+.

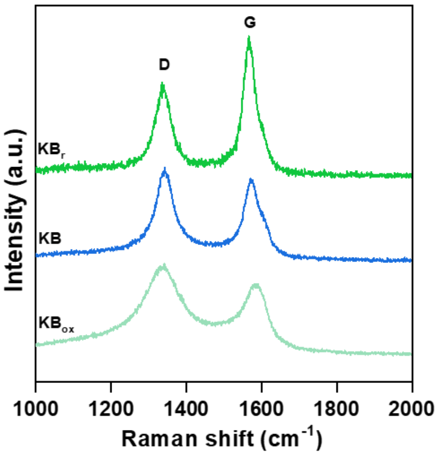

Figure S7. Raman spectra of KB, KB_{ox} and KB_r carbons with the graphitic G band (~1570 cm^{-1}) and disorder-induced D band (~1340 cm^{-1}).

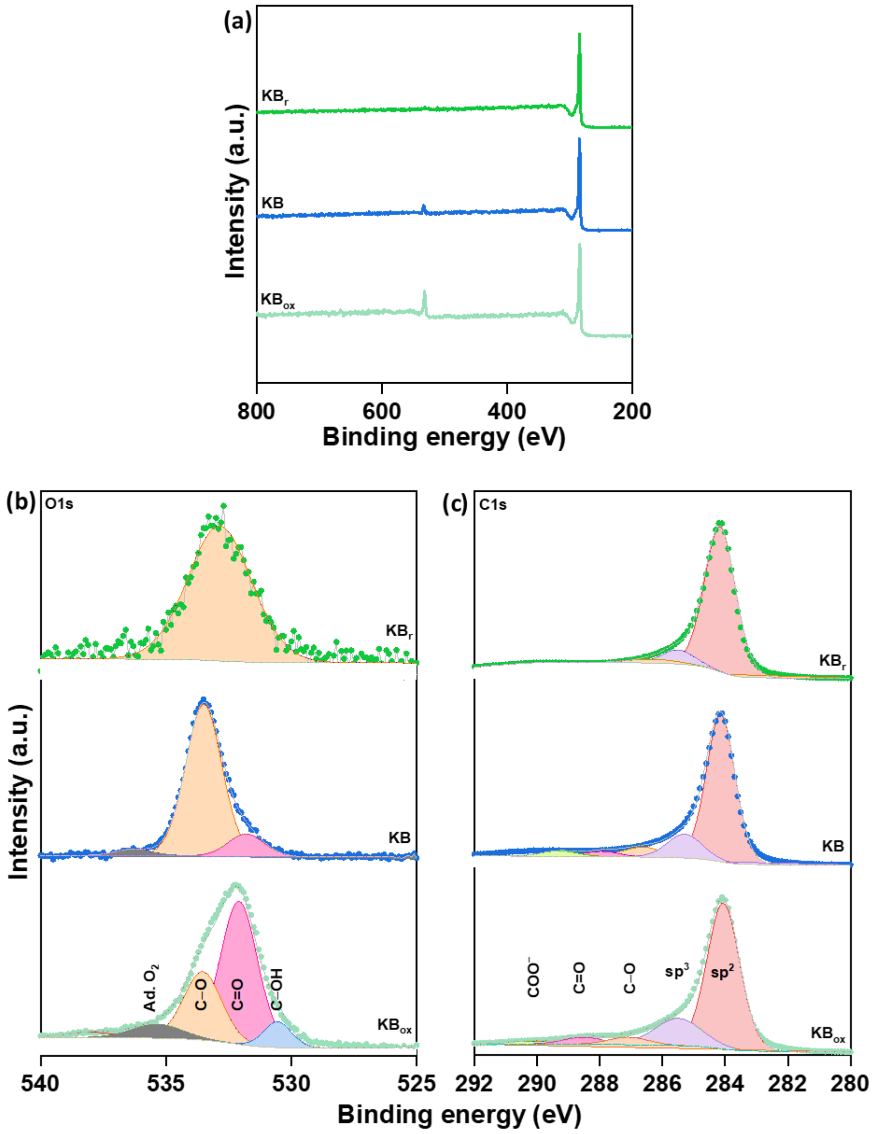

Figure S8. X-ray photoelectron spectra (XPS) of pristine and modified KB. (a) Wide scan (b) O1s and (c) C1s binding energy region.

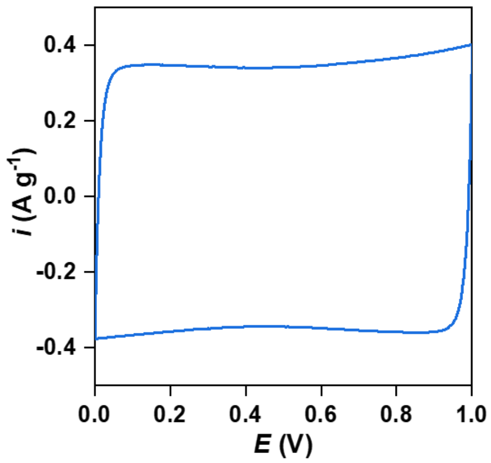

Figure S9. Cyclic voltammetry (400th cycle) of KB|separator|KB symmetric cell in the range of 0 to 1 V at 50 mV s^{-1} scan rate. The specific capacitance of KB electrode is calculated to be 12.7 F g^{-1} that gives an ECSA of 28 m^2 g^{-1}.

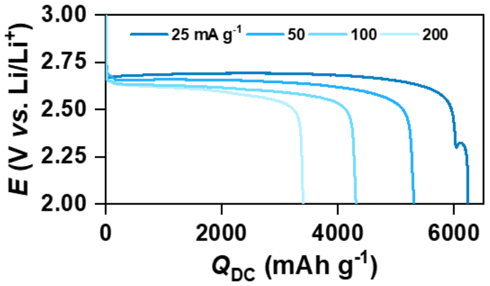

Figure S10. *E vs.* Q_{DC} plot for discharge of KB electrodes up to a cut-off potential of 2 V *vs.* Li/Li^+ in LiTFSI/TEGDME electrolyte (< 20 ppm H_2O) at different j_{DC}.

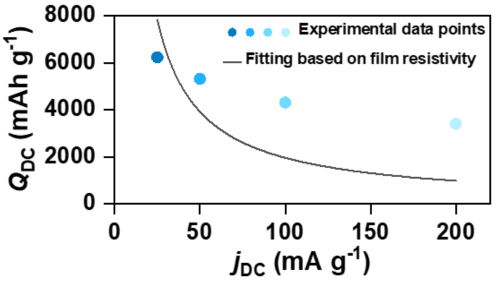

Figure S11. Q_{DC} *vs.* j_{DC} plot from Figure S10. Solid gray line represents the inverse relationship between Q_{DC} and j_{DC} based on the Li_2O_2 film resistivity.

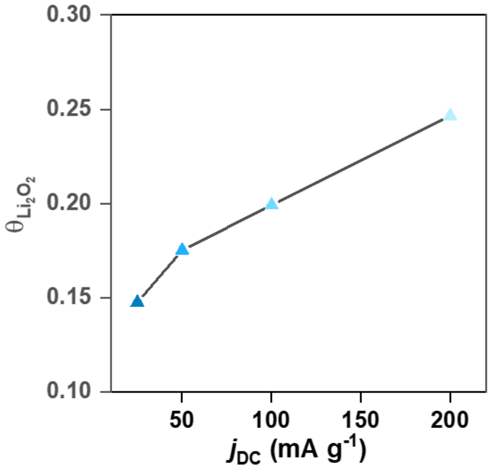

Figure S12. Plot of fraction of total electrode ($\theta_{Li_2O_2}$) covered by Li_2O_2 film of 15 nm thickness grown by surface reduction up to Q_{DC} of 1 mAh against j_{DC}.

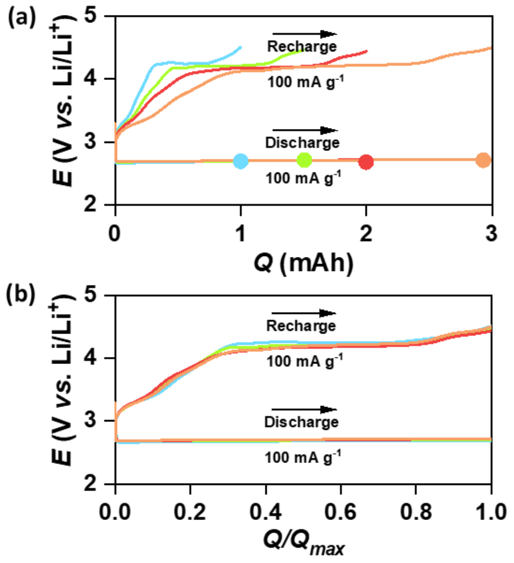

Figure S13. (a) E *vs.* Q plot for KB electrodes discharged up to different Q_{DC} of 1, 1.5, 2 and 3 mAh in LiTFSI/TEGDME electrolyte (< 20 ppm H_2O) at j_{DC} of 100 mA g^{-1}. (b) E *vs.* Q/Q_{max} plot from (a) by normalizing Q_{DC}.

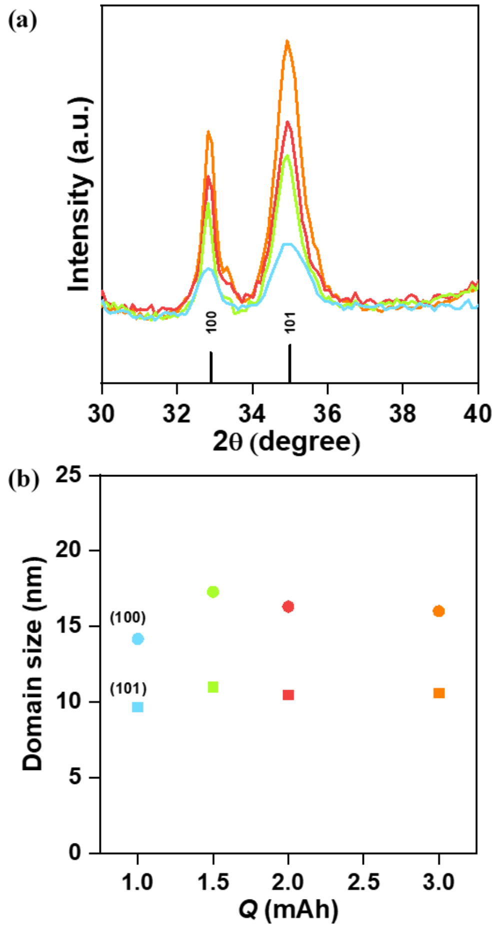

Figure S14. (a) XRD patterns of KB electrodes discharged up to Q_{DC} 1, 1.5, 2 and 3 mAh in LiTFSI/TEGDME electrolyte (< 20 ppm H_2O) at j_{DC} of 100 mA g^{-1}. All the XRD data are normalized to the intensity of the KB+CP peak at $2\theta = 54.3$. (b) Plot of domain size *vs*. Q_{DC} for both (100) and (101) reflections of Li_2O_2. Domain size is calculated by using Scherrer equation. XRD peak positions for standard Li_2O_2 sample are shown by black vertical lines at the bottom of (a).

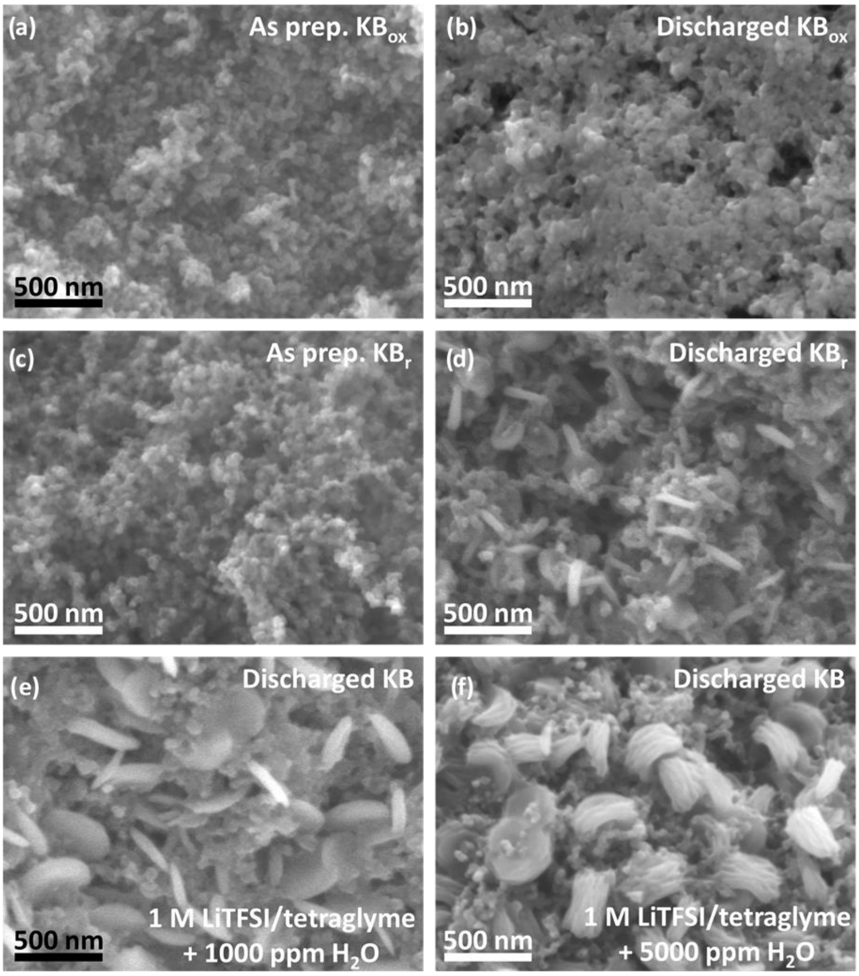

Figure S15. SEM images of (a) As prepared KB_{ox}, (b) discharged KB_{ox} up to Q_{DC} of 1 mAh at j_{DC} of 100 mA g^{-1}, (c) as prepared KB_r and (d) discharged KB_r up to Q_{DC} of 1 mAh at j_{DC} of 100 mA g^{-1} and KB discharged in LiTFSI/TEGDME electrolyte with (e) 1000 and (f) 5000 ppm H_2O up to Q_{DC} of 1 mAh at j_{DC} of 100 mA g^{-1}.

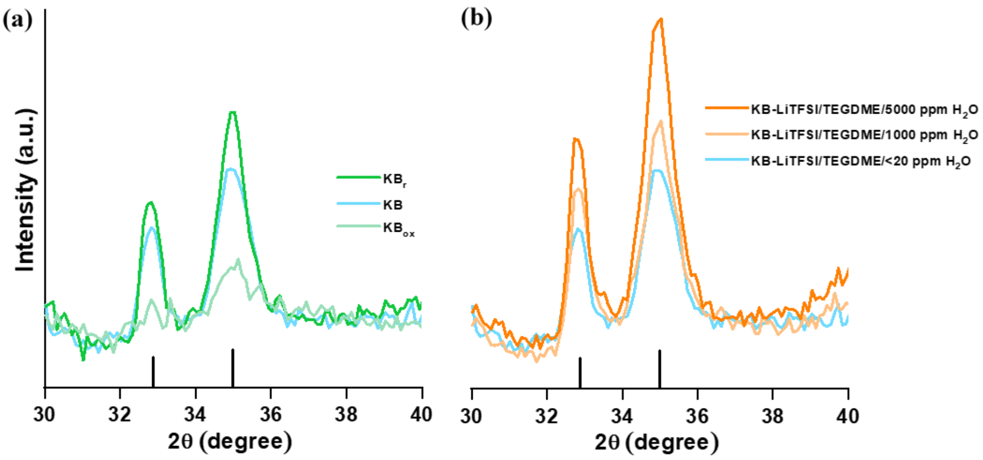

Figure S16. XRD patterns of discharged (a) pristine and modified KB electrodes in LiTFSI/TEGDME (< 20 ppm H_2O) electrolyte and (b) KB electrode in LiTFSI/TEGDME electrolyte with < 20, 1000 and 5000 ppm H_2O up to Q_{DC} of 1 mAh at j_{DC} of 100 mA g^{-1}. All the XRD data are normalized to the intensity of the KB+CP peak at 2θ = 54.3.

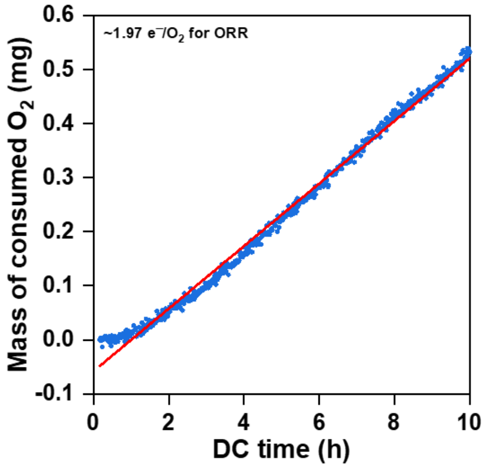

Figure S17. In situ measurement of mass of consumed O_2 during DC of KB electrode up to a Q_{DC} of 1 mAh in LiTFSI/TEGDME electrolyte (< 20 ppm H_2O) at j_{DC} of 100 mA g^{-1}. The red solid line represents a linear fit to the experimental data points. An ORR of 1.97 e^- /O_2 is calculated from the consumed O_2.

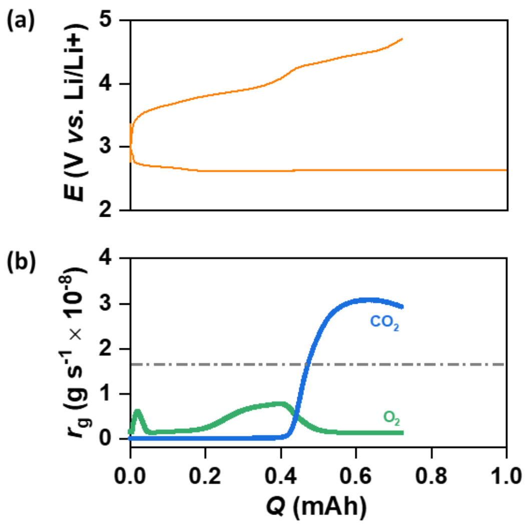

Figure S18. (a) *E vs. Q* plot for DC/RC of KB electrode at j_{DC} of 100 mA g^{-1} in LiTFSI/TEGDME electrolyte with 5000 ppm H_2O measured in a flow-type cell for online electrochemical mass spectrometry (OEMS) and (b) rate of gas evolution (r_g) during RC of the cell. The cut-off conditions for RC were set as 4.7 V *vs.* Li/Li^+ and 100% RC. The broken gray line in (b) represents the rate corresponding to $2e^-$ /O_2 process.

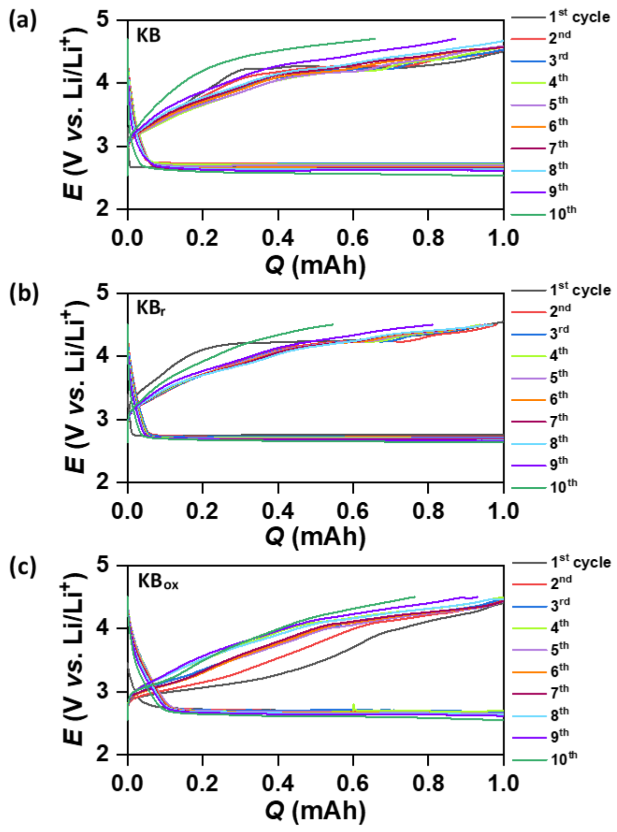

Figure S19. Galvanostatic cycles of (a) KB, (b) KB_r and (c) KB_{ox} electrodes for a fixed Q_{DC} of 1 mAh at j_{DC} of 100 mA g^{-1} in LiTFSI/TEGDME (< 20 ppm H_2O) electrolyte.

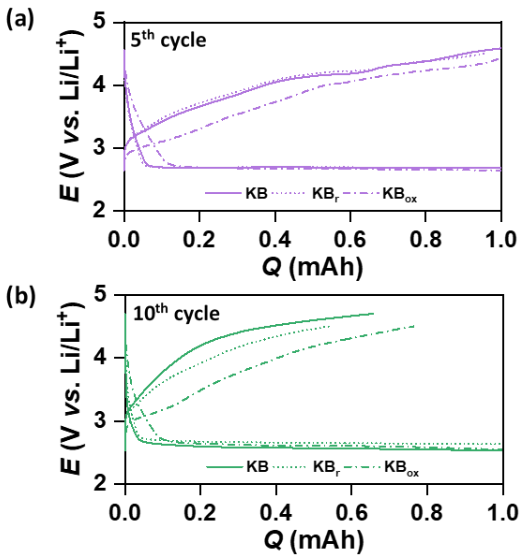

Figure S20. Comparison of KB, KB_r and KB_{ox} electrodes for the (a) 5th and (b) 10th galvanostatic cycles from Figure S19.

Table S1. Physico-chemical properties of KB, KB_{ox} and KB_r samples

Samples	O/C [XPS]	I_D/I_G [Raman]	ECSA $[m^2\ g^{-1}]$
**KB**	0.027	1.06	28
**KB_{ox}**	0.098	1.54	-
**KB_r**	0.003	0.71	-

References

[1] A. Dutta, K. Ito, Y. Kubo, *J. Mater. Chem. A* **2019**, *7*, 23199.

[2] Y. Holade, C. Morais, K. Servat, T. W. Napporn, K. B. Kokoh, *Phys. Chem. Chem. Phys*. **2014**, *16*, 25609.
